# Structure, mechanism, and inhibition of Hedgehog acyltransferase

**DOI:** 10.1016/j.molcel.2021.11.018

**Published:** 2021-12-16

**Authors:** Claire E. Coupland, Sebastian A. Andrei, T. Bertie Ansell, Loic Carrique, Pramod Kumar, Lea Sefer, Rebekka A. Schwab, Eamon F.X. Byrne, Els Pardon, Jan Steyaert, Anthony I. Magee, Thomas Lanyon-Hogg, Mark S.P. Sansom, Edward W. Tate, Christian Siebold

**Affiliations:** 1 Division of Structural Biology, Wellcome Centre for Human Genetics, University of Oxford, Roosevelt Drive, Oxford OX3 7BN, UK; 2 Department of Chemistry, Imperial College London, 82 Wood Lane, London W12 0BZ, UK; 3 Department of Biochemistry, University of Oxford, University of Oxford, South Parks Road, Oxford OX1 3QU, UK; 4 Structural Biology Brussels, Vrije Universiteit Brussel (VUB), Pleinlaan 2, 1050 Brussels, Belgium; 5 VIB-VUB Center for Structural Biology, Vlaams Instituut Biotechnologie (VIB), Pleinlaan 2, 1050 Brussels, Belgium; 6 National Heart and Lung Institute, Imperial College London, Exhibition Road, London SW7 2AZ, UK; 7 Department of Pharmacology, University of Oxford, Mansfield Road, Oxford OX1 3QT, UK

**Keywords:** Sonic Hedgehog signaling, Hedgehog acyl transferase, membrane-bound O-acyltransferase, heme, palmitoyl co enzyme A, cryo-EM structure, molecular dynamics simulations, integral membrane protein, small molecule inhibitor, drug

## Abstract

The Sonic Hedgehog (SHH) morphogen pathway is fundamental for embryonic development and stem cell maintenance and is implicated in various cancers. A key step in signaling is transfer of a palmitate group to the SHH N terminus, catalyzed by the multi-pass transmembrane enzyme Hedgehog acyltransferase (HHAT). We present the high-resolution cryo-EM structure of HHAT bound to substrate analog palmityl-coenzyme A and a SHH-mimetic megabody, revealing a heme group bound to HHAT that is essential for HHAT function. A structure of HHAT bound to potent small-molecule inhibitor IMP-1575 revealed conformational changes in the active site that occlude substrate binding. Our multidisciplinary analysis provides a detailed view of the mechanism by which HHAT adapts the membrane environment to transfer an acyl chain across the endoplasmic reticulum membrane. This structure of a membrane-bound *O*-acyltransferase (MBOAT) superfamily member provides a blueprint for other protein-substrate MBOATs and a template for future drug discovery.

## Highlights

• High-resolution cryo-EM structure of HHAT in complex with a SHH-mimetic megabody
• Heme is bound to HHAT Cys324 in a membrane cavity and is essential for function
• Palm-CoA binds in a central HHAT cavity adjacent to the catalytic histidine
• The competitive inhibitor IMP-1575 causes conformational changes in the active site

## Graphical abstract

**Figure:**
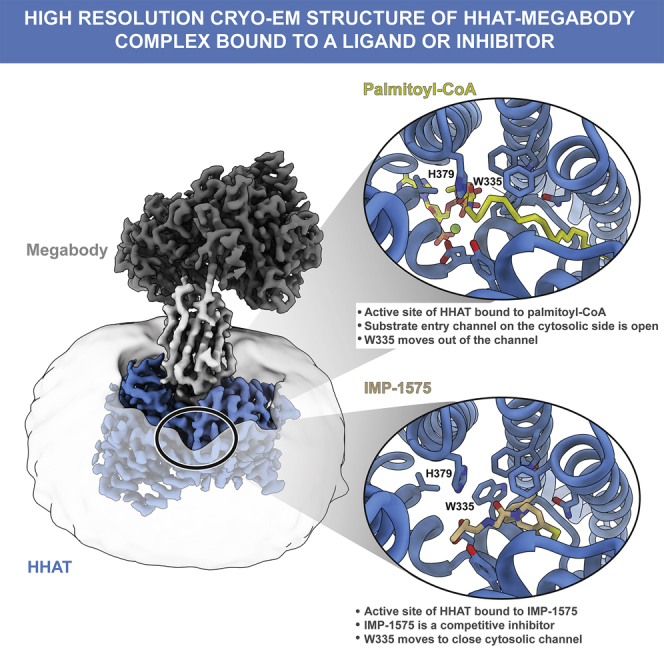

## Teaser

HHAT is a key enzyme in the Hedgehog signaling pathway and protein-substrate member of the membrane-bound *O*-acyltransferase (MBOAT) superfamily. Coupland et al. report the cryo-EM structures of HHAT bound to acyl-donor substrate, megabody, and inhibitor IMP-1575, providing insight into the structure-function relationship of HHAT, mechanism, and the basis for inhibition.

## Introduction

Hedgehog (HH) signaling is essential for human embryogenesis and stem cell regulation, and mutations in pathway members often cause congenital diseases, such as holoprosencephaly (Briscoe and Thérond, 2013; Ingham et al., 2011). Aberrant HH signaling is linked to the formation of numerous cancers, and it has been estimated that ∼25% of all fatal cancers show deregulated HH activation, making the pathway an important target for cancer drug discovery (Chahal et al., 2018; Wu et al., 2017). Post-translational protein lipidation is integral to HH signaling; the HH morphogen Sonic hedgehog (SHH) undergoes a highly unusual and unique maturation process in which a 45-kDa preprotein undergoes autocleavage and concomitant attachment of a cholesterol moiety at the new C terminus (Bumcrot et al., 1995; Porter et al., 1995, 1996). Following cleavage of a signal peptide, a palmitoyl (C16:0) acyl group is transferred to the amine of the new N-terminal cysteine (Cys24) in the endoplasmic reticulum (ER) (Pepinsky et al., 1998) to yield the mature 19-kDa, dually lipidated SHH signaling domain. HH acyltransferase (HHAT) catalyzes the irreversible transfer of a palmitoyl group from palmitoyl-coenzyme A (Palm-CoA) to SHH-Cys24 (Buglino and Resh, 2008; Chamoun et al., 2001), which is the rate-limiting step in HH ligand production. Mechanistically, it is unknown whether HHAT directly *N*-acylates HH proteins, or whether this occurs via *S*-acylation of Cys24 followed by *S*,*N*-acyl shift to yield the *N*-acylated product. Lipidation of SHH is required for interaction with its receptor Patched (PTCH1) at the signal-receiving cell surface (Kong et al., 2019), whereby the N-terminal palmitoyl group binds to PTCH1 and relieves inhibition of the G protein-coupled receptor Smoothened (SMO) to activate downstream HH signaling (Qi et al., 2018; Tukachinsky et al., 2016). Accordingly, HHAT knockout leads to a loss-of-function HH phenotype (Chen et al., 2004), and HHAT has been identified as a potential cancer drug target (Konitsiotis et al., 2014; Matevossian and Resh, 2015a; Petrova et al., 2013; Rodgers et al., 2016).

HHAT is a member of the membrane-bound *O*-acyltransferase (MBOAT) superfamily (Hofmann, 2000). The majority of MBOATs catalyze lipid chain transfer to small-molecule lipid substrates; however, HHAT, alongside porcupine acyltransferase (PORCN) and ghrelin *O*-acyltransferase (GOAT), acylates protein substrates. PORCN catalyzes *O*-palmitoleoylation of Wnt in development (Herr and Basler, 2012), while GOAT catalyzes ghrelin *O*-octanoylation in appetite regulation (Gutierrez et al., 2008; Yang et al., 2008). Structural data from bacterial and lipid-substrate MBOATs (Long et al., 2020; Ma et al., 2018; Qian et al., 2020; Sui et al., 2020; Wang et al., 2020) defined a common architecture, sharing an 8-transmembrane (TM) helix core domain that contains the active site, including a conserved histidine (Hofmann, 2000) and in the case of the lipid-substrate MBOATs, acyl-coenzyme A:cholesterol acyltransferase 1 (ACAT) and diacylglycerol *O*-acyltransferase 1 (DGAT), an acyl-coenzyme A substrate binding site. Topological studies suggested a similar domain for HHAT (Konitsiotis et al., 2015; Matevossian and Resh, 2015b). A recent structure of HHAT (Jiang et al., 2021) demonstrates that this is indeed the case, and provides intriguing insights into the likely mechanism of this complex protein-acylating enzyme. Here, we present a high-resolution cryogenic electron microscopy (cryo-EM) structure of HHAT in complex with the non-hydrolyzable Palm-CoA analog palmityl-CoA (nhPalm-CoA) and an inhibitory megabody. Our structural analysis reveals a cavity for SHH binding on the ER luminal face that leads to the active site. Surprisingly, we identified a heme molecule bound proximal to the Palm-CoA binding site that plays a crucial functional role. A further HHAT structure with a potent small-molecule inhibitor IMP-1575 bound identified significant conformational changes in the active site, which block Palm-CoA access to SHH. Our results clarify the structural mechanism by which an MBOAT family member can catalyze the acylation of a protein substrate while exchanging a lipid substrate across the ER membrane and provide a template for future development of a pharmacological inhibitor.

## Results

### Overall structure of human HHAT

To structurally and functionally characterize HHAT, we raised camelid antibodies (nanobodies [NBs]) against full-length human HHAT and isolated two NBs (NB169 and NB177) that bound to HHAT expressed in cells and in biolayer interferometry using purified HHAT with low nanomolar affinity (Figure S1). To overcome the technical hurdles of size and/or preferential orientation problems for cryo-EM structure analysis, we inserted the nanobodies into the *E. coli* K12 glucosidase YgjK scaffold resulting in ∼100 kDa megabodies (MBs; Uchański et al., 2021) (MB169 and MB177). We reconstituted the complex of human HHAT (incubated with nhPalm-CoA) and MB177 in detergent micelles and determined the cryo-EM complex structure to a 2.69-Å resolution using a localized refinement strategy (Figure S2). The high-quality cryo-EM map allowed the building of almost all side chains of HHAT and MB177 as well as nhPalm-CoA (Figure S2A).

**Figure 1:**
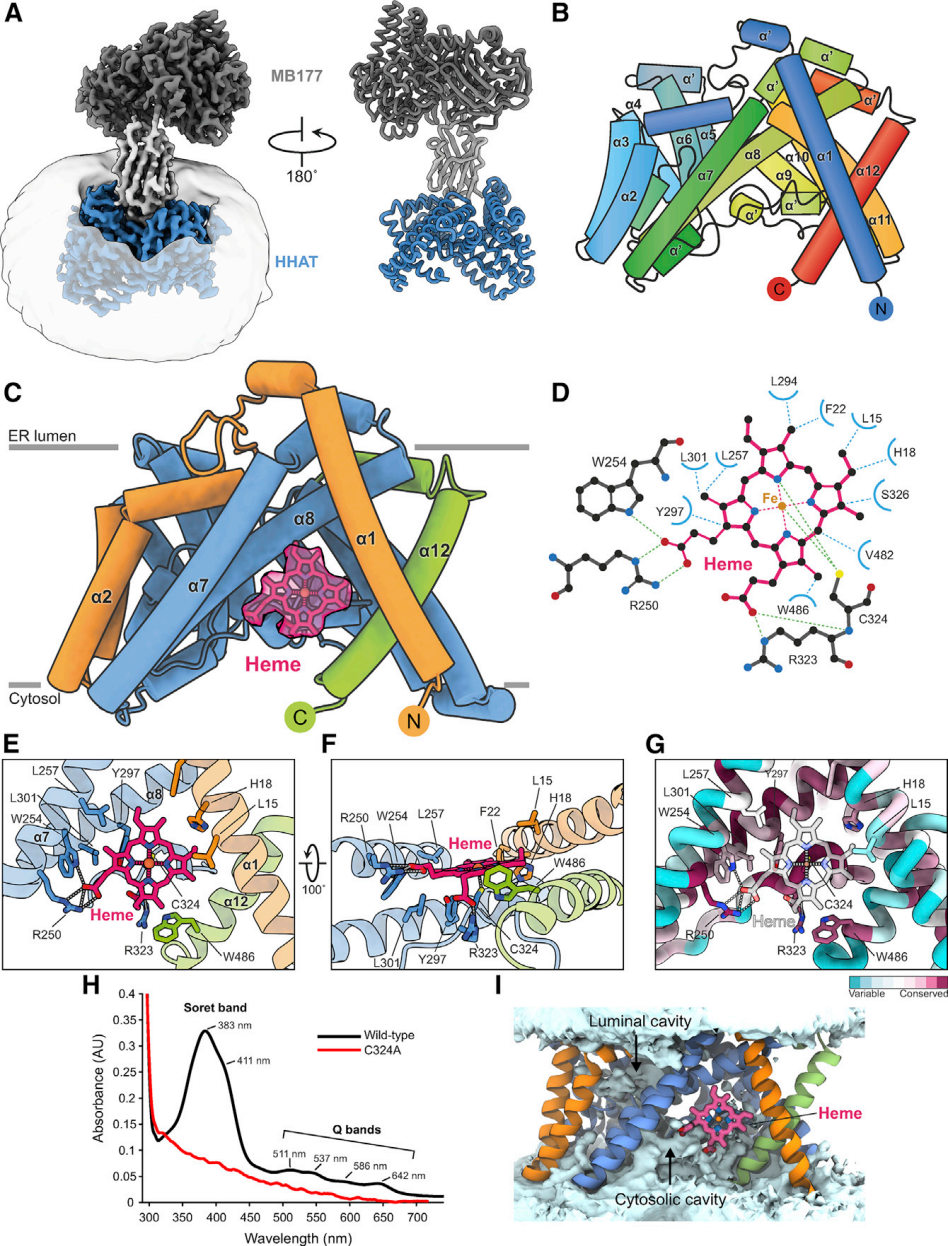
Structure of human HHAT (A) Cryo-EM map and ribbon representation of the HHAT-megabody 177 complex at 2.69-Å resolution. HHAT is represented in blue, the megabody scaffold in dark gray, and the nanobody in light gray. The detergent micelle is depicted. (B) Schematic of HHAT in rainbow coloring (blue: N terminus, red: C terminus). (C) Illustration of human HHAT. The structurally conserved MBOAT core is blue. The variable N (orange) and C (green) termini are highlighted. The heme-B group is depicted in magenta stick representation, with its cryo-EM map shown. (D) Schematic of the heme-B interactions with HHAT residues. (E–G) Close-up views of the HHAT heme-B binding site. (F) and (G) are colored as in (C). The sequence conservation (purple: conserved to green: variable) is mapped onto the structure in (G). (H) UV-vis spectra of HHAT wild-type (black) and HHAT-C324A (red). Wild-type HHAT shows the typical spectrum for a penta-coordinate thiolate Fe(III) heme with the split Soret band (at 383 and 411 nm) and the 4 Q bands (I: 642 nm, II: 586 nm, III: 537 nm, and IV: 511 nm). (I) Time-averaged solvent density (light blue surface) across 5 × 200 ns atomistic simulations of HHAT colored as in (C).

Our cryo-EM analysis revealed that HHAT forms a 1:1 complex with MB177 (Figure 1A; Video S1), which is consistent with size-exclusion chromatography-multiangle light scattering analysis (SEC-MALS) carried out in solution (Figure S1F). HHAT is composed of 12-TM helices with both termini located on the cytosolic side of the ER membrane (Figures 1B and S3). The 8-TM helix core of HHAT resembles that of the other MBOAT superfamily members (Figure 1C, colored blue). However, the HHAT N- and C-terminal regions are unique and distinct from other MBOAT structures (Figures 1C, colored orange and green, and S4). The terminal TM helices α1 and α12 cross over the top of the dimerization interface observed in DGAT and ACAT, thereby occluding this dimerization mode. In addition, TM helix α2 blocks the “lateral gate” from the lipid bilayer that is proposed to allow lipid substrate entry for DGAT and ACAT that mediate the attachment of fatty acids to lipid substrates (Long et al., 2020; Qian et al., 2020; Sui et al., 2020; Wang et al., 2020). Instead, the rearrangement of the N- and C-terminal helices results in the formation of a distinct pocket that is solvent accessible from the cytosolic side.

**Figure 2:**
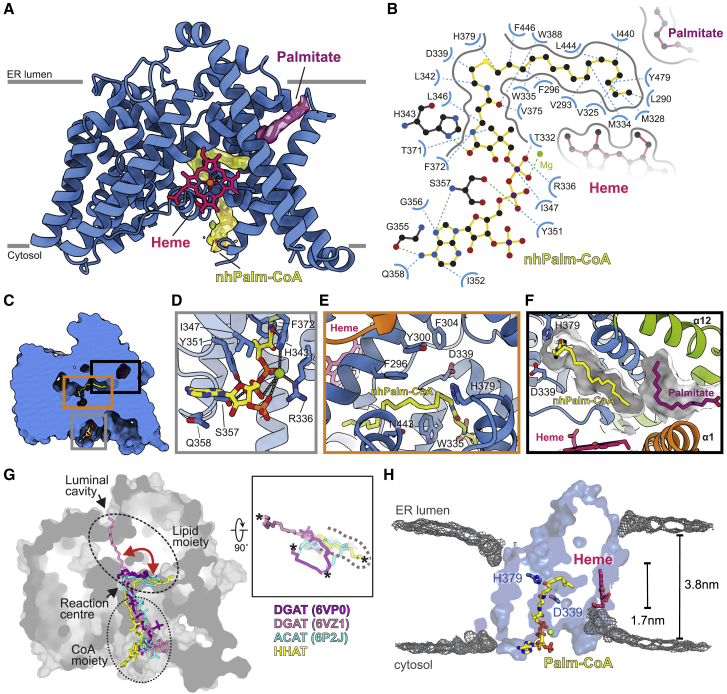
Structure of HHAT in complex with its co-factor analog nhPalm-CoA (A) Illustration of HHAT with bound nhPalm-CoA (yellow) and a palmitate molecule (purple) that forms part of the Palm-CoA binding site. The 2.69-Å resolution cryo-EM map for selected molecules is shown. (B) Detailed interactions of the Palm-CoA binding site. Hydrogen bonds are depicted in blue, the magnesium ion is in green. (C–F) Close-up views of the Palm-CoA binding site. (G) Structural superposition of HHAT with the DGAT-oleoyl-CoA complexes (PDB: 6VZ1 and 6VP0, respectively) and the ACAToleoyl-CoA (PDB: 6P2J) complexes, with only the acyl-CoA moieties depicted. The HHAT solvent-accessible surface is shown. Lipid and CoA moieties of the acyl-CoA substrates are circled. The red arrow indicates the conformational flexibility of the CoA substrate acyl chains. The close-up in the right panel highlights the different conformations of the acyl chains viewed from the ER luminal side. The HHAT palmitate binding pocket is marked with a dotted line. The terminal carbon atom of the acyl-CoA substrate is depicted with an asterisk for clarity. (H) Time-averaged density of lipid phosphate beads (gray isomesh) across 10 × 15-μs CG simulations of HHAT overlayed with the atomistic HHAT structure, colored as in (A). HHAT is depicted as a cut surface through the center of the protein to indicate the respective positions of the deformations.

**Figure 3:**
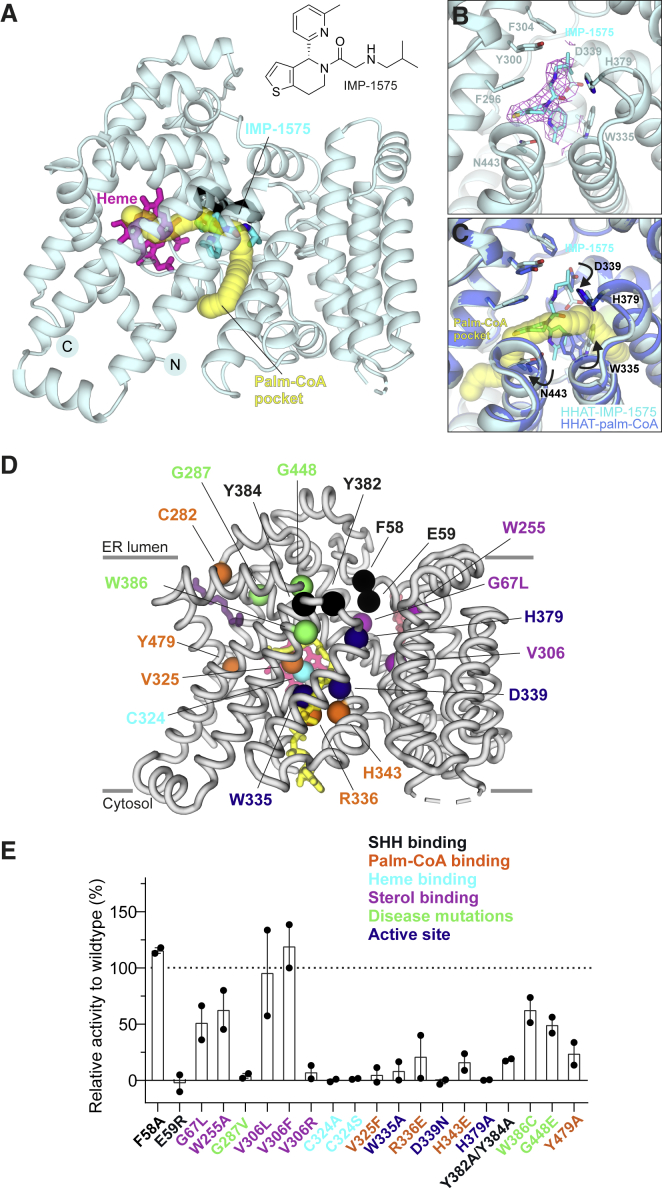
Structure of HHAT in complex with inhibitor IMP-1575 (A) Structure of HHAT in complex with the inhibitor IMP-1575. The Palm-CoA binding pocket calculated with the program CAVER is depicted in yellow, the bound heme moiety is in magenta, and IMP-1575 is highlighted in stick representation. View is rotated 90° around the x axis compared to Figure 1C. (B) Close-up view of the IMP-1575 binding site. IMP-1575 binds in the active site in close proximity to the catalytic His379. The cryo-EM map of the HHAT-IMP-1575 complex is shown as pink chicken wire. (C) Superposition of the HHAT-nhPalm-CoA (marine blue) and HHAT-IMP-1575 complexes (cyan). The Palm-CoA binding pocket is shown in yellow. The arrows indicate the conformational changes induced by inhibitor binding. IMP-1575 occludes Palm-CoA binding. Orientation is as in (B). (D and E) Structure-function analysis of HHAT mutants. A ribbon representation of HHAT with residues mutated in spheres is shown in (D). nhPalm-CoA (yellow), heme (pink), palmitate (dark purple), and cholesterol (red) are shown in stick representation. Activities of HHAT mutants relative to wild-type HHAT, measured by acyl-cLIP assay. n = 2 (quadruplicates), means ± SEMs between replicates; black dots are shown in (E) and represent means of replicates.

Video S1. Overview of 2.7-Å-resolution cryo-EM structure of the HHAT-MB177-nhPalm-CoA complex, related to Figures 1, 2, and 3

### HHAT is a heme protein

Surprisingly, we observed strong additional density in this solvent-accessible pocket that we identified as a heme-B molecule (Figure 1C). The major interaction is formed by the side chain of Cys324 that axially coordinates the Fe(III) ion of the heme-iron complex. Heme binding is supported by various conserved hydrophobic interactions (e.g., Leu257, Trp254, Trp486) and two salt bridges between HHAT residues Arg250 and Arg323 with the two carboxylate groups of the heme porphyrin ring (Figures 1D–1F). The heme binding site is highly conserved across HHAT homologs (Figures 1G and S3). To confirm the presence of an HHAT-bound heme moiety, we carried out UV-visible (UV-vis) spectroscopy (Figure 1H) and observed a spectrum typical of a cysteine-coordinated heme protein characterized by a broad Soret band ∼380 nm, followed by several weaker absorptions (Q bands) at higher wavelengths (from 500 to 650 nm) (Kühl et al., 2013; Uppal et al., 2016). HHAT-C324A, in which the cysteine that coordinates the heme iron is mutated to alanine, lacks the Soret and Q bands, further supporting the presence of an HHAT-heme complex observed in the structure. Cys324 was previously shown to be important for HHAT folding and suggested to be post-translationally modified in addition to other cysteine residues (Konitsiotis et al., 2015). The strong heme density on Cys324 is clearly distinct from the lipid-like attachments we observe for multiple cysteines in the HHAT structure (Figure S5), with partial density consistent with reversible long-chain *S*-acylation. Our discovery agrees with a recent structure of HHAT in complex with antibody fragments that also revealed a heme group in a similar conformation (Jiang et al., 2021) (Figures S6A and S6B).

The rearrangement of the termini forms a cytoplasmic cavity with the heme molecule at its apex, which is solvent accessible from the cytosolic side of the membrane in atomistic simulations (Figures 1I and S7). While visualization of the protein structure alone may suggest that the heme moiety would be embedded within the hydrophobic bilayer core, molecular dynamics (MD) simulations indicate that it is situated at the membrane-solvent interface, with the heme carboxyl groups puncturing the wetted cytoplasmic cavity. Thus, heme binding could be regulated by both membrane and cytoplasmic components.

### Mode of palmitoyl-CoA binding

We determined HHAT in complex with a non-hydrolyzable Palm-CoA analog (nhPalm-CoA) lacking the thioester carbonyl, which is bound within the HHAT core domain (Figures 2A and 2B; Video S1). The entire 16:0-CoA molecule is visible in the cryo-EM map. nhPalm-CoA binding occludes a continuous solvent cavity through HHAT that extends from the cytoplasmic cavity to the luminal face (Figure S7). The CoA adenine ring protrudes into the solvent at the cytosolic site, stacking between Ile352 and Gln358, and the N1 and N6 nitrogen atoms form hydrogen bonds with the amide nitrogen of Ser357 and the carbonyl oxygen of Gly355, respectively (Figures 2C and 2D). The CoA phosphate groups are coordinated by a metal ion, built as a magnesium ion based on coordination geometry (Harding, 2001), and several hydrogen bonds mediated by Arg336 and Ser357. The Palm-CoA moiety is shifted along the membrane normal in the HHAT binding site compared to its average simulated position in a membrane bilayer (Stix et al., 2020). This locates the Palm-CoA thioester in the core of the HHAT cavity close to the proposed reaction center residues His379 and Asp339 (Figure 2E) that have previously been shown to be important for the catalysis of palmitoyl transfer to SHH (Buglino and Resh, 2010; Hofmann, 2000). We observe a similar overall conformation of the co-factor and binding pocket when compared to the structure of HHAT bound to Palm-CoA (Jiang et al., 2021) (Figure S6C). However, some small conformational changes in the binding mode near the catalytic center are observed. We resolve a flip of the adenine base in the CoA moiety and a rotation of the pantothenic moiety that may be attributed either to chemical differences between the two Palm-CoA derivatives and/or differences in density interpretation between the two structures (Figure S6D).

The tail of the nhPalm-CoA 16:0 chain inserts into a binding pocket formed by rearrangement of the HHAT N- and C-terminal helices (Figures 2E and 2F). The palmitoyl moiety forms mainly hydrophobic interactions with residues located in the HHAT core; however, we also observe additional kinked lipid density that forms part of the palmitoyl substrate binding site, modeled as an additional palmitate moiety (Figure 2F). This arrangement of the termini restricts the acyl chain length to a maximum of 16 carbons and provides a rationale for previous analyses of SHH expression revealing acylation of the SHH N terminus predominantly by shorter acyl chains (e.g., myristate, myristoleate), in addition to palmitate, and to a far lesser extent by longer lipids (Long et al., 2015; Pepinsky et al., 1998). In contrast, the acyl-binding pocket of lipid MBOATs appears to be shallower and less defined, and the acyl chains show a high degree of conformational flexibility, even extending toward the luminal side (Figure 2G).

It has recently been postulated that Palm-CoA enters HHAT through an “archway” formed between α10 and α11 (Jiang et al., 2021). The authors observed extra cryo-EM density in this region, which they attribute to Palm-CoA (as deposited at the Electron Microscopy Data Bank under EMD-23836 and EMD-23837) (Figure S6E, left panel). To probe the stability of the modeled archway Palm-CoA, we performed atomistic MD simulations of the HHAT structure (5 × 100 ns) of Jiang et al. (2021) (PDB: 7MHY) bound to heme and with both Palm-CoA molecules present (Figure S6F). We observe a consistent displacement and dissociation of the archway Palm-CoA away from the modeled pose. Revisiting the cryo-EM density in this context reveals discontinuity of the acyl tail of the proposed archway Palm-CoA, which we suggest could instead be attributed to the glycol-diosgenin (GDN) detergent belt (Figure S6E, left panel). Our cryo-EM map also contains discontinuous densities in the same location (Figure S6F, right panel). Interestingly, we can identify an unambiguous density in our structure corresponding to the palmitoylation of Cys387 (Figure S5) that is located in the very place attributed to the acyl tail of the archway Palm-CoA by Jiang et al. (2021). Both structures have an extra cryo-EM density between α10 and α11 that resembles an acyl chain of ∼16 carbons length that we built as a palmitate in our model (Figure S6E). The density does not allow an unambiguous identification of the acyl molecule, and it is therefore plausible that a variety of lipids are able to bind in this cavity. The presence of the palmitoylation of Cys387 in one of our cryo-EM structures but not in others suggests that this site could be regulated via post-translational modification and that the lipidic composition of the membrane may influence the type of lipids binding to the cavity.

An intriguing question arising from HHAT function is how it may reduce the energetic barrier for palmitoyl transfer across the membrane. Coarse-grained (CG) MD simulations of HHAT in a model ER membrane revealed substantial bilayer deformation around 2 regions in opposing leaflets formed by Leu321-Thr327 (heme binding site) and around α helices 5 and 9 (Figures 2H, S7G, and S7H). These bilayer deformations funnel toward the reaction center, with a net reduction in bilayer width of up to ∼50% (1.7 nm) compared to the width at extended distances from the protein (3.8 nm). Thus, HHAT-induced bilayer thinning may reduce energetic barriers in the catalytic cycle and/or aid entry/exit of bound ligands.

### Structure of HHAT bound to small-molecule inhibitor IMP-1575

Given the importance of HHAT as a potential target to inhibit SHH signaling in disease, we determined the structure of HHAT bound to IMP-1575, the most potent HHAT small-molecule inhibitor reported to date (Lanyon-Hogg et al., 2021) (Figure 3). IMP-1575 binds in the middle of the reaction center, forming a hydrogen bond to the catalytic HHAT His379 and thus occluding Palm-CoA binding (Figures 3A–3C). Our previous computational analysis suggested that the stereogenic center of IMP-1575 has (*R*) absolute stereochemistry (Lanyon-Hogg et al., 2021), and this stereochemistry is confirmed in the cryo-EM structure. IMP-1575 binding leads to a rearrangement of the reaction center when compared to the HHAT-nhPalm-CoA complex, affecting the side chain conformations of the active site Asp339 as well as Asn443 and Trp335 (Figure 3C). Trp335 is situated in close proximity to the Palm-CoA thioester bond and exhibits a dramatic conformational switch between the two structures such that the side chain is rotated inward toward the Palm-CoA binding pocket when the inhibitor is bound (Video S1). This is consistent with kinetic studies of HHAT inhibition by IMP-1575 and rationalizes the mechanism of Palm-CoA competitive inhibition by IMP-1575 (Lanyon-Hogg et al., 2021). The active site rearrangements induced by IMP-1575 are similar to those of HHAT bound to a palmitoylated SHH peptide (Jiang et al., 2021) (Figure S6G), suggesting that IMP-1575 mimics the product-bound state of HHAT.

**Figure 4:**
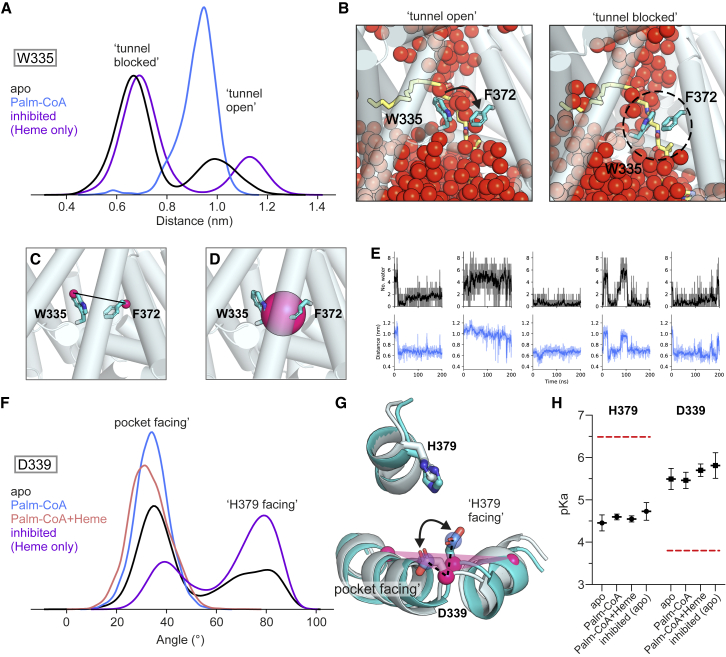
HHAT dynamics in simulations (A) Distance between Trp335-Cη2 and Phe372-Cα atoms in atomistic MD simulations of HHAT in the apo, Palm-CoA-bound, and inhibited/heme-only states (5 × 200 ns of each). (B) Snapshots showing the conformation of Trp335 at the start (“tunnel open”) and end (“tunnel blocked”) of a simulation of HHAT apo. Water atoms are shown as red spheres, overlaid with Palm-CoA (yellow sticks) to indicate the relative position within HHAT (transparent cylinders). (C and D) Close-up view of the Trp335-Cη2 to Phe372-Cα distance (C) and (D) a sphere (4 Å radius) positioned at the center of the geometry of Trp335/Phe372 Cα atoms, used to assess the Trp335 gating mechanism in (E). MDAnalysis was used to calculate the position of the sphere at each time point in the trajectory according to the updated center of geometry of the Trp335/Phe372 Cα. (E) Number of waters within the sphere (black) and Trp335/Phe372 distance (blue) versus time. The rolling mean is shown as an opaque line. (F–H) Dynamics of the HHAT reaction center. (F) Angle of the Asp339 side chain (defined as the angle between a vector formed by the Cα and Cγ atoms of Asp339 with respect to a plane formed by the Cα atoms of Met334, Asp339, and Leu346) across simulations of HHAT in apo, Palm-CoA-bound, Palm-CoA plus heme, and inhibited/heme states. (G) Overlay of the start (light cyan) and end (cyan) snapshots from a simulation of HHAT in the apo state. The angle between the side chain vector (blue) and plane (pink) is indicated. (H) The pKa of Asp339 and His379 across simulations of HHAT in the apo, Palm-CoA, Palm-CoA plus heme, and inhibited/heme-only simulations as calculated using pKa-traj. Red dashed lines indicate standard pKa values of histidine or aspartate residues. Error bars indicate the standard error of the mean between replicates.

To further probe Trp335 dynamics, we performed atomistic MD simulations of HHAT. When the Palm-CoA binding site was unoccupied (starting from both the Palm-CoA- or IMP-1575-bound structures), the Trp335 indole ring moved toward Phe372 to close the Palm-CoA tunnel (Figures 4A–4E). This conformation poses a hydrophobic restriction in the Palm-CoA binding pocket. In contrast, when Palm-CoA was bound, Trp335 remained in an upright conformation. Thus, water permeation through HHAT is prevented by either Palm-CoA or Trp355 movement. In simulations, we also observe dynamic behavior of the Asp339 side chain, which faces toward either Gly341/Leu342 (“pocket facing”) or the solvated catalytic core (“His379 facing”). Palm-CoA binding restricts Asp339 to the pocket facing conformation due to steric occlusion of the His379 facing conformation (Figures 4F and 4G). We estimated the pKa of Asp339/His379 across the simulations (Figure 4H), yielding a mean pKa of ∼5.6 for Asp339 and of ∼4.6 for His379. The pKa values are shifted due to a large number of proximal hydrophobic residues and, crucially, the donor/acceptor pairing is reversed, suggesting that these residues do not function in a proton shuttle mechanism. The ability of His379 to form an H-bond with IMP-1575, along with proximity to the Palm-CoA reaction center and the shifted pKa, may suggest a role acting as a H-bond donor in activation of the Palm-CoA carbonyl in catalysis.

### Structure-activity analysis to identify key features for HHAT function

To investigate the role of the observed structural features in catalysis, we generated and purified a series of structure-guided HHAT mutants. These were evaluated for enzymatic activity by monitoring palmitoylation of a FAM-labeled substrate peptide (**CGPGRGFGKR**(K-FAM)G-CONH_2_) in real time using the acylation-coupled lipophilic induction of polarization (acyl-cLIP) assay (Lanyon-Hogg et al., 2019) (Figures 3D and 3E). Mutation of the canonical MBOAT active site residues Asp339 and His379 (D339N and H379A) abolishes activity, as does mutation of heme-binding Cys324 (C324A and C324S). The distance (∼19 Å) and lack of a clear connection between the heme Fe ion and the reaction center argues against a direct role in catalysis and supports a suggested role of Cys324 in HHAT folding (Konitsiotis et al., 2015). The mutation of Trp335 to alanine (W335A) also reduced HHAT activity, consistent with its proposed role as a gatekeeper residue of the Palm-CoA pocket. Modification of the phosphate/magnesium binding site for nhPalm-CoA (R336E and H343E) at the cytosolic site completely abrogated activity, similar to V325F, which was designed to block the Palm-CoA fatty acid binding tunnel. This is also seen for Y479A, which interferes with shielding the acyl chain of the nhPalm-CoA from direct contact with the heme group. This confirms the observed Palm-CoA binding site in our structure and shows that proper Palm-CoA binding is essential for HHAT function, as expected.

**Figure 5:**
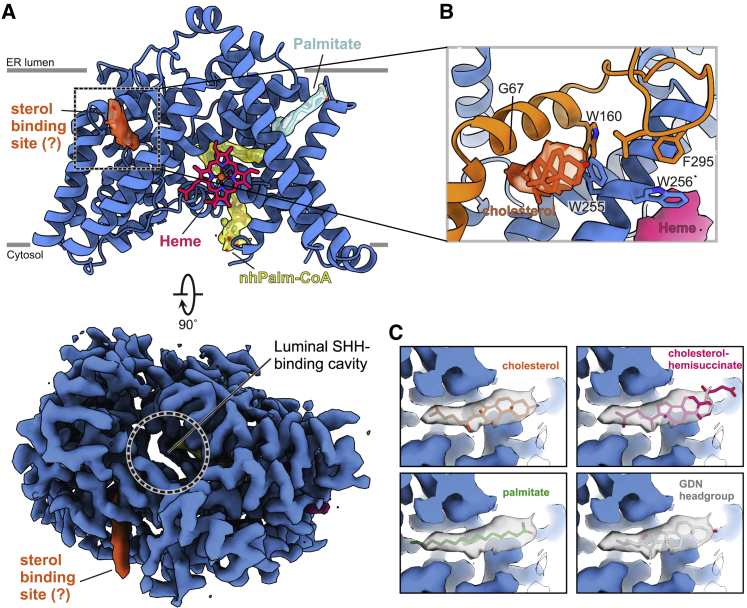
A potential sterol-binding site adjacent to the luminal SHH binding cavity (A) Illustration of HHAT with selected lipids and substrates highlighted and depicted and drawn with the final cryo-EM map. View and color coding is as in Figure 2. (B) Close-up view of the potential sterol-binding site. The final cryo-EM (orange) is suggestive of a cholesterol or similar sterol molecule. (C) Comparison of the different lipids fitted into the sterol map shown in (B).

On the luminal face, Phe58 and Glu59 have been suggested to interact with the incoming SHH substrate (Jiang et al., 2021). We found that whereas modification of Phe58 (F58A) did not markedly affect activity, a charge reversal at E59R leads to a complete loss of activity, presumably due to repulsion of the positively charged character of the SHH N-terminal peptide substrate. Moreover, alanine exchange of the tyrosine residues located above the reaction center (Tyr382 and Tyr384), and thus likely interacting with SHH, significantly reduces HHAT activity. We also investigated the importance of an observed sterol binding site that is located adjacent to the luminal SHH-binding cavity, and which has Val306 at its center and is lined by Gly67 and Trp255 (Figure 5). Mutation of Val306 to hydrophobic residues (V306L and V306F) is tolerated, but the introduction of a positive charge (V306R) severely reduces enzymatic activity (Figure 3E). G67L and W255A show some effects; however, they still retain ∼50% activity, presumably either by maintaining packing or allowing a sterol molecule to bind. Our structure-guided mutagenesis analysis supports the structure features observed in the cryo-EM map and identifies key regions important for HHAT function.

Finally, we tested several known disease-associated HHAT mutations (Figures 3D and 3E). The G287V mutation was reported to abrogate catalytic activity and induce syndromic 46,XY disorder of sex development (Callier et al., 2014). Our results confirm this, and our structure suggests that this mutation would severely distort the packing of helix α8 and the N-terminal helix α1, disrupting the heme binding site and potentially rearranging the palmitoyl binding tunnel and/or reaction center. Mutation W386C has been implicated in intellectual disability (Agha et al., 2014) and shows a minor reduction in enzymatic activity, according to our data. Our structure has clear density for a palmitic acid moiety in this area, and thus a mutation to cysteine may lead to palmitoylation at this position. The G448E mutation was identified in a melanoma cell line and may play a role in cancer development (Kawakami et al., 2001). Our structure shows that it is located at the rim of the luminal access cavity for SHH. Even though our enzymatic data show only a modest decrease in activity, we hypothesize that its effect could be more profound on palmitoylation of full-length SHH. A glutamate residue at this position would protrude straight into the access channel, potentially blocking the larger protein from entering the HHAT active site. We also attempted to express the L257P mutant, which causes microcephaly (Abdel-Salam et al., 2019), but expression levels were too low to isolate the protein in useful quantities (data not shown). Leu257 is located in helix α7 and forms direct hydrophobic contacts to the heme group (Figure 1F). We hypothesize that mutation to proline not only would be disruptive to heme binding but also cause a helix kink that could disrupt the core MBOAT fold, leading to misfolded protein. In summary, our data provide a mechanistic rationale for observed disease-linked mutations in HHAT.

### A luminal access cavity for SHH

**Figure 6:**
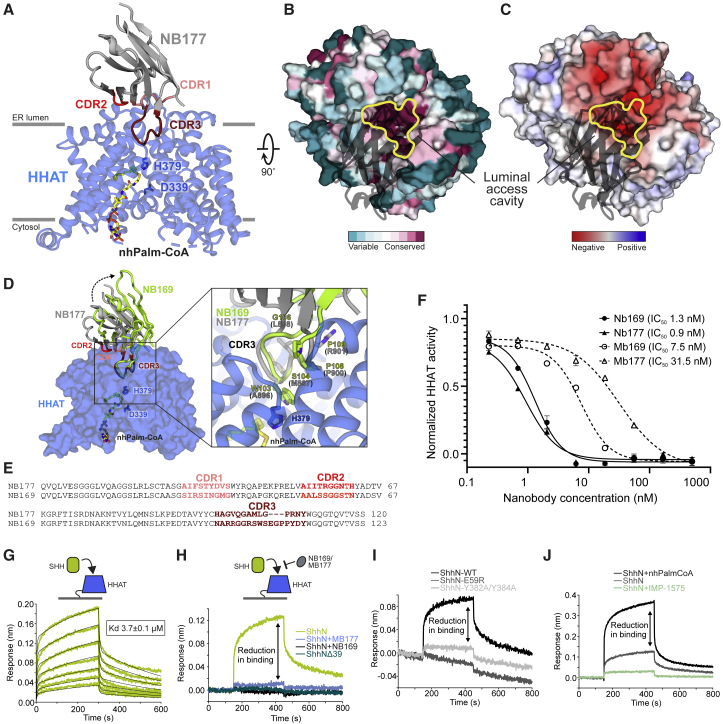
A luminal access cavity for SHH (A) Illustration of the HHAT-MB177 complex. HHAT is blue, MB177 is gray, with the complementary determining regions (CDRs) colored salmon (CDR1), red (CDR2), and dark red (CDR3). Active site HHAT residues His379 and Asp339 are highlighted in stick representation. The active site entry tunnel, which is mainly blocked by MB177-CDR3, protrudes deeply into HHAT. (B and C) Surface representation of HHAT, with MB177 shown in black ribbon. Sequence conservation (B) and electrostatic potential (C) (from ±8 kT/eV) are mapped onto the solvent-accessible HHAT surface. The active site entry tunnel is outlined in yellow. Orientation is 90° rotated around the y axis. (D) Structure of the HHAT-MB169 complex. HHAT is shown as a blue surface and NB169 is shown in green. NB177 is shown superimposed and colored as in (A) and (B) (using HHAT as template). The orientation is as in (B). A close-up of CDR3 is shown in the box, revealing the close structural similarity of the Cα backbone. (E) Sequence alignment of nanobodies NB177 and NB169 with the CDRs colored as in (A). The overall sequence identity is 77.4%, with only 27.8% identity in the CDRs (CDR1: 22.2%, CDR2: 40.0%, CDR3: 23.5%). (F) Acyl-cLIP assay to test SHH palmitoylation. Dose-response curves of nanobodies and megabodies inhibiting SHH palmitoylation by HHAT. The IC_50_ of the nanobodies is below the detection limit of the assay ([HHAT] ≈10 nM). n = 1 (triplicates), means ± SEMs. (G) Multicycle kinetics measurement of the ShhN-HHAT interaction with real-time sensorgrams for different ShhN concentrations. (H) ShhN binding is abolished in a SHH construct missing the first 15 residues, or in the presence of MB169 and MB177. (I and J) HHAT mutants E59R and Y382A/Y384A, located in the luminal binding cavity, cannot bind SHH (I), and SHH binding is reduced by 50% in the absence of Palm-CoA and completely abolished in the presence of inhibitor IMP-1575 (J).

Our structural analyses show that the lipid substrate entry site in the membrane in other MBOATs is occluded by rearrangement of the HHAT termini (Figure S4). How does SHH, a hydrophilic protein substrate, access the HHAT active site? Our structure reveals a distinct luminal cavity arrowing toward the catalytic His379 in the reaction center, where the major HHAT interaction loop of MB177 (CDR3 loop) is located, suggesting an access path for SHH (Figure 6A). This cavity is highly conserved in all HHAT family members (Figure 6B) and forms part of a larger acidic interface spanning the luminal surface (Figure 6C). This negative patch complements the positively charged N terminus of human SHH (C24-GPG**R**GFG**KRRH**P**KK**), the first six residues of which are essential for HHAT-mediated palmitoylation (Hardy and Resh, 2012). This N-terminal stretch is disordered in structures of SHH in complex with the human hedgehog-interacting protein (HHIP) (Bishop et al., 2009; Bosanac et al., 2009 ), cell adhesion molecule-related/downregulated by oncogenes (CDO) (McLellan et al., 2008), or apo SHH (Hall et al., 1995), and is only visible in the SHH-PTCH1 complex structure where the N-terminal SHH palmitate protrudes into the PTCH1 core (Qi et al., 2018; Rudolf et al., 2019).

We also determined the structure of HHAT in complex with another MB (MB169), which shares the same epitope and complementarity-determining region (CDR) loop conformation, despite belonging to a different NB family (sequence identity of CDR regions <28%) (Figures 6D and 6E). In these structures, the CDR3 loops of MB169 and MB177 penetrate the luminal cavity, binding in close proximity to the active H379 (∼5-Å distance). Both NBs and corresponding MBs are efficient inhibitors of SHH palmitoylation, inhibiting palmitoyl transfer to a SHH peptide with half-maximal inhibitory concentration (IC_50_) values of 1.3 nM (NB169), 0.9 nM (NB177), 7.5 nM (MB169), and 31.5 nM (MB177) (Figure 6F). This suggests that the NBs and SHH compete for access to the active site via the luminal cavity. To further test this hypothesis, we set up a competition binding assay using biolayer interferometry (BLI). The full-length N-terminal signaling domain of SHH (ShhN) only lacking the lipid attachments bound HHAT with a K_d_ of 3.7 μM (Figure 6G), whereas an SHH truncation, missing the 15 N-terminal residues essential for palmitoylation (ShhNΔ39) was not able to bind HHAT (Figure 6H). When carrying out the same experiment in the presence of a constant concentration of 200 nM NB169 or MB177, ShhN binding was occluded, confirming that the luminal cavity of HHAT is the access route for SHH (Figure 6H). To support our competition binding experiments, we tested HHAT mutants E59R and Y382A/Y384A, both located at the apex of the luminal cavity, in our BLI assay (Figure 6I). Both mutants severely impaired SHH binding, in agreement with our activity assay showing no (for E59R) or reduced (for Y382A/Y384A) palmitoylation. Our data are also supported by the recently determined cryo-EM structure of HHAT bound to its product palm-SHH peptide (Jiang et al., 2021) that also highlights the importance of HHAT E59 as well as Y382 and Y384 for interaction with SHH. Interestingly, the ShhN-HHAT interaction was reduced ∼3-fold in the absence of nhPalm-CoA and almost completely abolished in the presence of IMP-1575 (Figure 6J), suggesting that SHH binding is dependent on Palm-CoA binding to HHAT, likely due to Palm-CoA-mediated reorganization of Trp335 and/or Asp339.

## Discussion

Cysteine-bound heme-B iron complexes in prototypical heme proteins (e.g., hemoglobin, myoglobin, cytochrome P450) play key roles in oxygen storage, transfer, and activation, as well as being mediators of electron transfer. We identified a heme-B bound to HHAT and showed that mutation of the heme-binding cysteine results in loss of enzymatic activity. Although the cytoplasmic cavity where the heme molecule is bound shows conservation in other MBOAT family members, the position of Cys-324 appears to be unique to HHAT. Could the HHAT-bound heme group have a regulatory role or is it present to block dimerization observed in other small-molecule lipid MBOATs? A possibility could be that the heme-Cys324 binding site is a regulatory modification site that can compete with, for example, Cys-palmitoylation, as it has previously been shown that the C324A mutant affects the availability of *S*-palmitoylation sites within HHAT (Konitsiotis et al., 2015). Crosstalk between palmitoylation and heme attachment has not previously been reported, but would provide an appealing additional level of regulation, as has been shown between palmitoylation and ubiquitination/phosphorylation in TM proteins (Tian et al., 2008; Yount et al., 2012).

HHAT has been proposed to possess a transporter function for Palm-CoA (Asciolla and Resh, 2019). Our structural analysis reveals a distinct Palm-CoA binding site essential for acyl transfer that would not allow for a direct transport mechanism. However, we cannot rule out that when HHAT is in an apo state (without SHH or NB bound in the luminal cavity), a conformational change could occur that would allow the CoA palmitoyl chain to adopt an elongated conformation pointing toward the luminal side. Such different conformations of the acyl moiety were previously observed in the structures of DGAT (Figure 2G), and further structural studies of HHAT apo state(s) will be required to determine the mechanism of a potential transport function.

**Figure 7:**
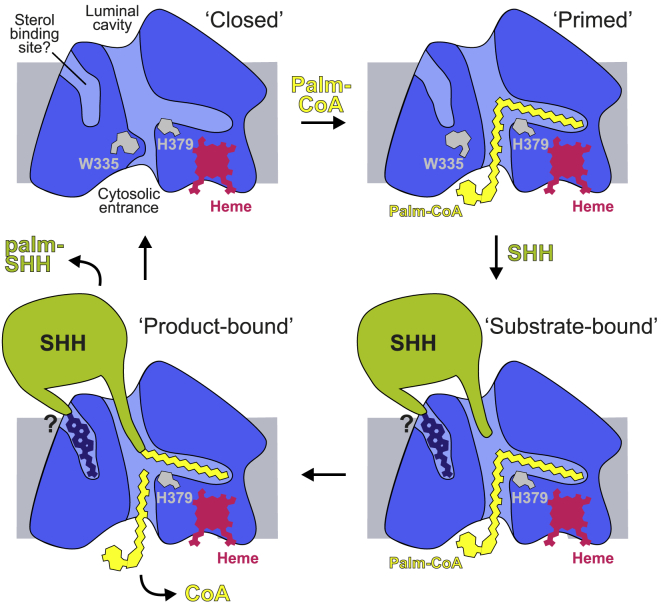
Model for HHAT-mediated palmitoylation of SHH Without any substrate bound, HHAT adopts a “closed” conformation, with Trp335 blocking the Palm-CoA-binding pocket (top left panel). Binding of Palm-CoA from the cytosolic side leads to a rearrangement of the active site involving residues His379 and Asp339 and primes HHAT for SHH binding (top right panel). The SHH N terminus binds HHAT from the luminal cavity to access the active site (bottom right panel). SHH binding may be supported by binding the C-terminal SHH-cholesterol moiety to a potential sterol-binding pocket located adjacent to the luminal cavity. Once acyl transfer to SHH occurs, palmitoylated SHH detaches from HHAT via either lateral exit into the membrane and/or supporting secreted and membrane proteins (e.g., Scube2, Dispatched) (bottom left panel).

We propose a model of HHAT function based on our structures (Figure 7). In the absence of substrate, Trp335 blocks water permeation by obstructing the central solvent channel (Figure 4). The lipid tail of Palm-CoA enters from the cytosolic face and Trp335 rotates into an “open” conformation. This HHAT “priming” allows the SHH N terminus to bind inside the luminal cavity, and this binding is occluded by MB169 and MB177, which compete with SHH for HHAT binding (Figure 6H). We observe a larger density present in a luminal pocket of HHAT adjacent to the luminal cavity, consistent with a sterol moiety (Figure 5). It is interesting to note, given the C-terminal cholesterylation unique to hedgehog homologs across all known proteins, that a cholesterol group could occupy the lipid density. Conserved Asp339 may form a charge interaction with the SHH N-terminal amine to orient the residue for attack; this residue is Asn in PORCN and GOAT, which acylate internal residues that are more likely to be oriented by complementary hydrogen bonding. It remains unclear whether *N*-palmitoylation is directly catalyzed by HHAT, or whether it results from spontaneous rearrangement of a thiol-attached intermediate (Mann and Beachy, 2004). Palmitoylation will increase the hydrophobicity of an already C-terminally cholesterylated SHH protein. Acylated SHH is therefore unlikely to exit HHAT directly into the ER lumen and needs to detach via lateral exit into the membrane, and/or supported by secreted and membrane proteins (e.g., Scube2, Dispatched). This requirement for assisted product release is supported by our previous observation that *N*-palmitoylated SHH or SHH N-terminal peptide is a relatively potent inhibitor of HHAT under cell-free conditions (Lanyon-Hogg et al., 2019).

Inhibiting HH signaling is a proven strategy in anticancer therapy. Most prior drug development has focused on developing inhibitors of the G protein-coupled receptor (GPCR) and HH signal transducer SMO, and SMO inhibitors are used in the clinic for the treatment of patients with basal cell carcinoma, medulloblastoma, and acute myeloid leukemia (Wu et al., 2017). However, patients frequently develop mutations in SMO that cause drug resistance. Here, we provide a structural template for the development of HHAT inhibitors, a validated alternative strategy to inhibit SHH signaling. Moreover, our structure suggests approaches to control lipid attachment to SHH, which could be used to engineer SHH morphogens with different specificities to manipulate stem cells and morphogen gradients.

### Limitations of the study

The findings presented here offer crucial insights into the initial Palm-CoA loading step in the HHAT enzymatic cycle and into inhibition of the reaction center for future development of therapeutic agents. However, important questions about the HHAT mechanism will need to be addressed in future studies. A comprehensive understanding of the entire HHAT mechanism requires elucidation of intermediate conformations within the catalytic cycle via structural methods, alongside corresponding energetic profiles, such as can be obtained using quantum mechanical/molecular mechanics (QM/MM) methods. Discerning these intermediate structures will require astute deployment of advancing cryo-EM methods, such as the use of conformation-specific NBs/MBs to trap scarce protein subpopulations and/or the use of small-molecule mimetics of reaction intermediates. QM/MM calculations are limited by the size of the simulated system, making application to enzymes acting on complex lipidic substrates computationally challenging (Medina et al., 2018).

Determination of the structure of HHAT bound to its substrate SHH may also assist in the interpretation of densities protruding into HHAT, including whether the proposed sterol-binding pocket (Figure 6) could be occupied by the C-terminal cholesteryl moiety of SHH. One prevailing question relates to whether palmitoylated-SHH exits HHAT either by moving laterally into the surrounding membrane or via “upward” dissociation into the luminal solvent. A first glimpse of a putative product exit process is offered by simulations in which we observe spontaneous and stable binding of a lipid molecule between helices α5 and α9 of HHAT, whereby one lipid acyl-tail mimics the proposed position of palmitoylated-SHH in the reaction center (Figure S8; Video S1). Binding of the product mimetic lipid-tail is accompanied by outward movement of α5, which correlates with inward swivel of Tyr377 to open the luminal gate (Figures S8A–S8G). Removal of lipid from the gate region or via pulling the lipid through the gate enabled relaxation of the α5–α9 distance, accompanied by reset and outward swivel of the Tyr377 side chain (Figures S8F–S8H). These data offer tantalizing clues to the potential mechanism of palmitoyl-SHH unloading (Figure S8I) into the membrane, which will be investigated in future experiments. Product exit studies will also need to account for whether the amine group of SHH-Cys24 is directly palmitoylated or whether the reaction proceeds via initial *S*-palmitoylation followed by *S*,*N-*acyl rearrangement. Thus, our current studies point the way from structure determination to an atomistic mechanistic understanding of HHAT function in a complex cell membrane environment. Finally, future studies should translate these mechanistic insights into a cellular context and the impact on SHH maturation and signaling.

## STAR★Methods

### Key resources table

**Table:** 

REAGENT or RESOURCE	SOURCE	IDENTIFIER
**Antibodies**		

Mouse anti-Rho [1D4] monoclonal IgG	University of British Columbia	N/A
Goat anti-mouse, horseradish peroxidase(HRP)-conjugate polyclonal IgG	Merck	Cat# A0168; RRID: AB_257867
Rabbit anti-His monoclonal IgG(25B6E11)	Genscript	Cat# A01857
Goat anti-rabbit, Alexa Fluor 555 polyclonal IgG	Life Technologies	Cat# A21429; RRID: AB_2535850
Streptavidin-HRP conjugate	Sigma-Aldrich	Cat# GERPN1231

**Bacterial and virus strains**		

BL21-DE3	NEB	Cat# C2527I
DH5α	Invitrogen	Cat# 18263012
WK6	Zell and Fritz, 1987	N/A

**Chemicals, peptides, and recombinant proteins**		

Dulbecco’s Modified Eagle’s Medium, high glucose	GIBCO	Cat# 21969-035
FreeStyle 293 Expression Medium	GIBCO	Cat# 12338026
Fetal Bovine Serum	Life Technologies	Cat# 10270106
L-Glutamine	GIBCO	Cat# 25030081
MEM non-essential amino acids	GIBCO	Cat# 11140050
Trypsin-EDTA (0.25%), phenol red	Thermo-Fisher	Cat# 25200056
D-biotin	Sigma-Aldrich	Cat# B4639
Polyethylenimine, branched	Sigma-Aldrich	Cat# 408727
Polybrene infection reagent (10 mg/mL stock = 1,000 × )	Merck	Cat# TR-1003-G
Doxycycline hydrocholoride	Merck	Cat# D3447
Valproic Acid (sodium salt)	Cayman Chemicals	Cat# 13033
Pyrobest DNA Polymerase	Takara	Cat# R005A
QuikChange site-directed mutagenesis Kit	Agilent	Cat# 200517
Quick Ligation Kit	NEB	Cat# M2200S
cOmplete protease inhibitor cocktail	Sigma-Aldrich	Cat# 11697498001
Phosphatase inhibitor cocktail	Sigma-Aldrich	Cat# P8340
Phenylmethylsulfonyl fluoride (PMSF)	Melford	Cat# P20270
1D4 peptide “TETSQVAPA”	Genscript	Peptide TETSQVAPA
Cholesteryl Hemisuccinate Tris Salt (CHS)	Anatrace	Cat# CH210
n-Dodecyl-β-D-Maltopyranoside (DDM)	Anatrace	Cat# D310
Glyco-diosgenin (GDN)	Anatrace	Cat# GDN101
Octyl Glucose Neopentyl Glycol (OGNG)	Anatrace	Cat# NG311
1-Palmitoyl-2-Oleoyl-sn-Glycero-3-Phosphocholine (POPC)	Anatrace	Cat# P516
CYMAL-7 Neopentyl Glycol	Anatrace	Cat# NG327
16:0 Ether Coenzyme A (palmityl-CoA, nhPalm-CoA)	Avanti Lipids	SKU 870740P
IMP-1575	This manuscript	N/A
Lysozyme	Sigma-Aldrich	Cat# L6876
Benzonase	Merck-Millipore	Cat# 70664
Isopropyl β-d-1-thiogalactopyranoside (IPTG)	FluoroChem	Cat# M02726
Dithiothreitol (DTT)	Melford	Cat# D11000
Tris(2-carboxyethyl)phosphine hydrochloride (TCEP)	Sigma-Aldrich	Cat# C4706
Bovine serum albumin	Sigma-Aldrich	Cat# A4503
Triton X-100	Merck	Cat# 1086431000
16% Formaldehyde solution	Thermo-Fisher	Cat# 28906
VECTASHIELD Antifade Mounting Medium with DAPI	Vector Laboratories Inc.	Cat# H-1200
4x Laemmli Sample Buffer	Bio-Rad	Cat# 1610747
InstantBlue Coomassie protein stain	Abcam	Cat# ISB1L
Palmitoyl-Coenzyme-A	Sigma-Aldrich	Cat# P9716
FAM-SHH peptide ‘CGPGRGFGKR (K-FAM)G-CONH2′	Lanyon-Hogg et al., 2019	Synthesized in-house as previously described (Imperial College London)
3C protease, His-tagged	Purified from BL21 cells transformed with pET28-3C protease plasmid	N/A

**Critical commercial assays**		

Vectastain Elite ABC kit	Vector laboratories	Cat# PK-7100, RRID:AB_2336827
NeuroTrace 435/455 Blue Fluorescent Nissl Stain	Invitrogen	Cat# N21479
Proximity ligation assay - Duolink *in situ* red starter kit mouse/RA	Sigma-Aldrich	Cat# DUO92101
Click-It EdU Cell proliferation kit for imaging, Alexa Fluor 555 dye	Thermo-Fisher	Cat# C10338

**Deposited data**		

Coordinates of the HHAT-MB169-nhPalm-CoA complex	This manuscript	PDB ID 7Q70
Cryo-EM density map of the HHAT-MB169-nhPalm-CoA complex	This manuscript	EMD-13861
Coordinates of the HHAT-MB177-nhPalm-CoA complex	This manuscript	PDB ID 7Q1U
Cryo-EM density map of the HHAT-MB177-nhPalm-CoA complex	This manuscript	EMD-13764
Coordinates of the HHAT-MB177-IMP1575 complex	This manuscript	PDB 7Q6Z
Cryo-EM density map of the HHAT-MB177-IMP1575 complex	This manuscript	EMD-13860
Original images of the HHAT IF experiments shown in Figure S1	This manuscript	https://doi.org/10.17632/23gtbdhdwf.1

**Experimental models: cell lines**		

HEK293S GnTI^−/−^	ATCC	Cat# CRL-3022; RRID: CVCL_A785
HEK293T	ATCC	Cat# CRL-3216; RRID: CVCL_0063
COS-7	ATCC	Cat# CRL-1651; RRID: CVCL_0224
HEK293T Lenti-X	Takara/Clontech	Cat# 632180

**Recombinant DNA**		

Plasmid: pHR-CMV-TetO2-HHAT-wildtype-1D4	This manuscript	N/A
Plasmid: pHR-CMV-TetO2-HHAT-wildtype-Avi-1D4	This manuscript	N/A
Plasmid: pHR-CMV-TetO2-HHAT-wildtype-mVenus-1D4	This manuscript	N/A
Plasmid: pHR-CMV-TetO2-HHAT-F58E-Avi-1D4	This manuscript	N/A
Plasmid: pHR-CMV-TetO2-HHAT-E59R-Avi-1D4	This manuscript	N/A
Plasmid: pHR-CMV-TetO2-HHAT-G67L-Avi-1D4	This manuscript	N/A
Plasmid: pHR-CMV-TetO2-HHAT-W255A-Avi-1D4	This manuscript	N/A
Plasmid: pHR-CMV-TetO2-HHAT-G287V-Avi-1D4	This manuscript	N/A
Plasmid: pHR-CMV-TetO2-HHAT-V306L-Avi-1D4	This manuscript	N/A
Plasmid: pHR-CMV-TetO2-HHAT-V306F-Avi-1D4	This manuscript	N/A
Plasmid: pHR-CMV-TetO2-HHAT-V306R-Avi-1D4	This manuscript	N/A
Plasmid: pHR-CMV-TetO2-HHAT-C324A-Avi-1D4	This manuscript	N/A
Plasmid: pHR-CMV-TetO2-HHAT-C324S-Avi-1D4	This manuscript	N/A
Plasmid: pHR-CMV-TetO2-HHAT-V325F-Avi-1D4	This manuscript	N/A
Plasmid: pHR-CMV-TetO2-HHAT-W335A-Avi-1D4	This manuscript	N/A
Plasmid: pHR-CMV-TetO2-HHAT-R336E-Avi-1D4	This manuscript	N/A
Plasmid: pHR-CMV-TetO2-HHAT-D339N-Avi-1D4	This manuscript	N/A
Plasmid: pHR-CMV-TetO2-HHAT-H343E-Avi-1D4	This manuscript	N/A
Plasmid: pHR-CMV-TetO2-HHAT-H379A-Avi-1D4	This manuscript	N/A
Plasmid: pHR-CMV-TetO2-HHAT-Y382A/Y384A-Avi-1D4	This manuscript	N/A
Plasmid: pHR-CMV-TetO2-HHAT-W386C-Avi-1D4	This manuscript	N/A
Plasmid: pHR-CMV-TetO2-HHAT-G448E-Avi-1D4	This manuscript	N/A
Plasmid: pHR-CMV-TetO2-HHAT-Y479A-Avi-1D4	This manuscript	N/A
Plasmid: pMESy4-NB169-His_6_-EPEA	This manuscript	N/A
Plasmid: pMESy4-NB177-His_6_-EPEA	This manuscript	N/A
Plasmid: pMESP23E2-MB169-His_6_	This manuscript	N/A
Plasmid: pMESP23E2-MB177-His_6_	This manuscript	N/A
Plasmid: pET22B-hSHH(39-193)-3C-His_6_	Whalen et al., 2013	N/A
Plasmid: pET22B-hSHH(24-193)-3C-His_6_	Whalen et al., 2013	N/A
Plasmid: pHR-CMV-TetO2-HA-BirA-cytoplasmic	This manuscript	N/A
Plasmid: pMD2.G	Addgene, gift from Didier Trono	Addgene plasmid #12259
Plasmid: psPAX2	Addgene, gift from Didier Trono	Addgene plasmid #12260
Plasmid: pET28-3C-protease	Robinson et al., 2021	N/A
Plasmid: pUC19	Sigma-Aldrich	Cat# OGS590

**Software and algorithms**		

SnapGene Software	SnapGene/Insightful Science	https://www.snapgene.com/
CLC Genomics Workbench 12	QIAGEN	https://www.qiagen.com/
ImageJ	Schneider and Sheldrick, 2002	https://imagej.nih.gov/ij/download.html
Clustal Omega	Sievers and Higgins, 2018	https://www.ebi.ac.uk/Tools/msa/clustalo/
GraphPad Prism	GraphPad Software	https://www.graphpad.com/scientific-software/prism/
ASTRA 6	Wyatt	https://www.wyatt.com/products/software/astra.html
EPU	FEI	https://www.thermofisher.com/us/en/home/electron-microscopy/products/software-em-3d-vis/epu-software.html
RELION 3.1	Zivanov et al., 2018	https://www3.mrc-lmb.cam.ac.uk/relion/index.php/Main_Page
cryoSPARC	Punjani et al., 2017	https://cryosparc.com
UCSF Chimera	Goddard et al., 2007	https://www.cgl.ucsf.edu/chimera/download.html
Phenix	Afonine et al., 2018	https://phenix-online.org/download/
PDBsum	Laskowski et al., 2018	http://www.ebi.ac.uk/thornton-srv/databases/pdbsum
Coot	Emsley and Cowtan, 2004	https://www2.mrc-lmb.cam.ac.uk/personal/pemsley/coot/
PyMOL	Schrodinger LLC, 2010	https://pymol.org/2/
Consurf	Ashkenazy et al., 2010	https://consurf.tau.ac.il/2016/
MolProbity	Davis et al., 2007	http://molprobity.biochem.duke.edu/
ForteBio Data Analysis 11.1	ForteBio/Sartorius	http://www.sartorius.com/en
TraceDrawer	Ridgeview Instruments	https://tracedrawer.com/
MATLAB (R2021a)	The MathWorks	https://www.mathworks.com
Caver 3.0 PyMol Plugin	Chovancova et al., 2012	https://caver.cz
propKa	Søndergaard et al., 2011	https://github.com/jensengroup/propka-3.0
propKaTraj	Becksteinlab, propkatraj 1.1.0	https://github.com/Becksteinlab/propkatraj
VMD 1.9.2	Humphrey et al., 1996	http://www.ks.uiuc.edu/Research/vmd/
martinize.py	de Jong et al., 2013	https://github.com/cgmartini/martinize.py
insane.py	Wassenaar et al., 2015	http://www.cgmartini.nl/images/tools/insane/insane.py
MemProtMD	Newport et al., 2019	https://memprotmd.bioch.ox.ac.uk
CG2AT	Vickery and Stansfeld, 2021	https://github.com/owenvickery/cg2at
MDAnalysis	Michaud-Agrawal et al., 2011	https://www.mdanalysis.org
GROMACS	Hess et al., 2008	www.gromacs.org
Modeler	Fiser and Sali, 2003	https://salilab.org/modeller/
Martini 2.2	de Jong et al., 2013	http://cgmartini.nl/
Python 3.6	Open Source software	https://www.python.org
UCSF ChimeraX	Goddard et al., 2018	https://www.rbvi.ucsf.edu/chimerax/

**Other**		

TALON Superflow Metal Affinity Resin	Clontech	Cat# 635668
Ni-NTA Agarose	QIAGEN	Cat# 30210
CNBr-Activated Sepharose 4B	GE Healthcare	Cat# GE17-0430-01
0.45 μm sterile PES syringe filters	VWR	Cat# 514-0075
HiLoad 16/600 Superdex 200 pg	GE Healthcare	SKU GE28-9893-35
HiLoad 16/600 Superdex 75 pg	GE Healthcare	SKU GE28-9893-33
Superose 6 Increase 10/300 GL	GE Healthcare	SKU GE29-0915-96
Vivaspin 20, 100,000 MWCO PES	Sartorius	Cat# VS2042
Vivaspin 500, 100,000 MWCO PES	Sartorius	Cat# VS0141
Amicon Ultra-15 Centrifugal Filter Unit	Merck-Millipore	Cat# UFC903096
PP Microplate, 96-well, black	Greiner	Item no. 655209
Streptavidin (SA) Biosensors	ForteBio/Sartorius	Mfg. reference 18-0009
QUANTIFOIL Holey Carbon Cu 300	Quantifoil	Cat# X-101-Cu300
Round filter paper for Vitrobot	Agar Scientific	Cat# 47000-100
UV-STAR microplate, 96-well	Greiner	Item no. 655801
384-well assay plate	Corning	Cat# 3575
12% Mini-PROTEAN TGX Precast Protein Gels	Bio-Rad	Cat# 4561046
4-15% Mini-PROTEAN TGX Precast Protein Gels	Bio-Rad	Cat# 4561086
ForteBio Octet Red 96e	ForteBio/Sartorius	N/A
CLARIOstar	BMG Labtech	N/A
EnVision Xcite 2104	PerkinElmer	Cat# 2105-0010
ImageQuant LAS 4000	GE Healthcare	N/A

### Resource availability

#### Lead contact

Further information and requests for reagents should be directed to and will be fulfilled by the Lead Contact Christian Siebold (christian@strubi.ox.ac.uk).

#### Materials availability

Plasmids generated in this study will be made available on request, but we may require payment and/or a completed Materials Transfer Agreement if there is potential for commercial application.

### Experimental model and subject details

#### Cell lines

All cell lines used in this study are listed in the Key resources table and were cultured under standard growth conditions (37°C, 5-8% CO_2_). HEK293T were used for transient mammalian expression using pHLsec vector (Aricescu et al., 2006). HEK293T Lenti-X cells were utilized to generate lentiviruses with the pHR-CMV-TetO_2_ vector (Elegheert et al., 2018), which were subsequently used to infect HEK293S cells and produce stable cell lines. Both cell lines were maintained in Dulbecco’s Modified Eagle’s Medium (DMEM high glucose, GIBCO) supplemented with L-glutamine, MEM non-essential amino-acids (NEAA; both GIBCO) and 10% fetal bovine serum (FBS, Life Technologies) with no addition of antibiotics. Cells were grown and maintained in standard T75 (75 cm^2^ - LentiX) or T175 (175 cm^2^ - HEK293T) flasks. Cos-7 cells were used for immunofluorescence staining experiments (transfected with pHR-CMV-TetO_2_ vectors). Cos-7 cells were grown in complete medium consisting of DMEM supplemented with 10% (v/v) FBS, L-Glutamine and NEAA. For immunofluorescence staining experiments, fixed Cos-7 cells were incubated with nanobodies in PBS. We used *E. coli* DH5α competent cells for cloning and *E. coli* BL21-DE3 for expression of ShhN. *E. coli* WK6 cells were used for nanobody and megabody expression.

### Method details

#### HHAT expression and purification

For protein expression, DNA encoding wild-type HHAT from *Homo sapiens* (VAR_024743; UniprotKB: Q5VTY9) was cloned into pHR-CMV-TetO_2_ vector (Elegheert et al., 2018) in frame with a C-terminal Rho1D4 antibody epitope tag (Molday and MacKenzie, 1983) (TETSQVAPA) alone (pHR-CMV-TetO2-HHAT-1D4), preceded by monoVenus (pHR-CMV-TetO2-HHAT-mVenus-1D4) or biotinylation tag (GLNDIFEAQKIEWHE) (pHR-CMV-TetO2-HHAT-Avi-1D4). For production of HHAT mutants, the HHAT gene was cloned into the pUC19 cloning plasmid and point mutations were introduced by QuikChange site-directed mutagenesis (Agilent) and then transferred into pHR-HHAT-Avi-1D4.

For expression, stable cell lines were created as described (Elegheert et al., 2018). HEK293S Lenti-X producer cells (Takara/Clontech) were transfected in DMEM with 2% (v/v) FBS with 12 μg of pHR-CMV-TetO2 construct vector alongside 11 μg envelope plasmid (pMD2.G; Addgene #12259) and 11 μg packaging plasmid (psPAX2; Addgene #12260) using polyethylenimine (PEI) at 2.5:1 (w/w) ratio to total DNA. After 2 days, cell culture media containing viral particles was filtered, supplemented with 1 μg/mL polybrene and added to HEK293S GnTI- TetR cells. For biotinylated samples, media containing viral particles for HHAT constructs was mixed in a 50:1 (v/v) ratio with media from Lenti-X cells transfected as above with biotin ligase (BirA) in a pHR-CMV-TetO_2_ vector and then added to HEK293S cells. These cells were incubated 37°C, 8% CO_2_ for at least 2 passages before expansion or freezing and transfer to cryo-storage. Stably-transfected HEK293S cell cultures were grown in 2-10 L suspension at 37°C, 130 rpm, 8% CO_2_ in Freestyle 293 media, supplemented with 1% (v/v) FBS, 1% L-glutamine and NEAA (GIBCO) for at least 2 passages. Cells at 2-3 × 10^6^ cells/mL were induced with 50 ng/mL doxycycline (Merck) and 5 mM valproic acid (Cayman Chemicals). For biotinylated proteins, 0.8 mM biotin was added the day preceding induction. Media was harvested after 72 h, centrifuged (Beckman Avanti, 1500 x g, 10 min, 4°C) and pellets were flash-frozen and stored at −80°C.

For purification of HHAT for cryo-EM, all steps were performed at 4°C. Cells were lysed and membranes solubilised in 20 mM Tris pH 8.0, 300 mM NaCl, 5% (v/v) glycerol, 1 mM phenylmethylsulfonyl fluoride (PMSF), with 1% (v/v) membrane protease inhibitors (Sigma-Aldrich) and 1.5% (w/v) Octyl Glucose Neopentyl Glycol (OGNG) 40:1 cholesteryl hemisuccinate (CHS) (both Anatrace) by 90 min stirring. Cell debris was removed by centrifugation (Beckman Avanti, 45 min, 40,000 x g, 4°C) and supernatants were diluted 3-fold in 20 mM Tris pH 8.0, 300 mM NaCl, 5% (v/v) glycerol, before incubation 2 h with 500 μL CNBr-activated Sepharose beads (GE Healthcare) coupled to monoclonal Rho-1D4 antibody (University of British Columbia) per 1 g pellet. Beads were collected on a flow column and washed with 20 column volumes (CV) 20 mM Tris pH 8.0, 300 mM NaCl (wash buffer, WB) with 0.522% (w/v) OGNG 40:1 CHS, then 20 CV WB with 0.174% (w/v) OGNG 40:1 CHS, 20 CV WB with 0.06% (w/v) glyco-diosgenin (GDN) (Anatrace) and 20 CV WB with 0.02% (w/v) GDN. HHAT was eluted by overnight incubation with 500 μM 1D4 (TETSQVAPA) peptide (Genscript).

Purified megabody was added at 1:1.5 molar ratio and 0.05% (w/v) CYMAL-7-NG and 0.01 mg/mL 1-palmitoyl-2-oleoyl-sn-glycero-3-phosphocholine (POPC) (both Anatrace) were added before concentration in Vivaspin 100,000 kDa molecular weight cut-off (MWCO) centrifugal concentrators (Sartorius) to a volume of ∼500 μL. The sample was passed over a Superose 6 Increase 10/300 GL column (GE Healthcare), equilibrated in 10 mM Tris pH 8.0, 150 mM NaCl, 0.02% (w/v) GDN. Protein-containing fractions were pooled and concentrated to 4 mg/mL.

Biotinylated HHAT and HHAT mutants were purified in a similar manner to above. Membranes were solubilised in 20 mM HEPES pH 7.5, 300 mM NaCl, 5% (v/v) glycerol, 1 mM PMSF, with 1% (v/v) membrane protease inhibitors and 1.0% (w/v) n-dodecyl-β-D-maltopyranoside (DDM) 40:1 CHS (Anatrace). After centrifugation, supernatants were diluted 2-fold in 20 mM HEPES pH 7.5, 300 mM NaCl, 5% (v/v) glycerol, before incubation 2 h with 100 μL Rho-1D4 antibody-coupled Sepharose beads per 1 g pellet. Beads were washed with 25 column volumes (CV) 20 mM HEPES pH 7.5, 300 mM NaCl (wash buffer, WB) with 0.09% (w/v) DDM 40:1 CHS, then 75 CV WB with 0.03% (w/v) DDM 40:1 CHS. HHAT was eluted by overnight incubation with 500 μM 1D4 (TETSQVAPA) peptide (Genscript). Protein was flash-frozen and stored at −80°C before direct use in biolayer interferometry or enzyme activity assays.

For HHAT samples for SEC-MALS, HHAT was purified in a similar manner to the biotinylated samples from cells expressing the pHR-HHAT-mVenus-1D4 construct. Membranes were solubilised in 20 mM HEPES pH 7.5, 300 mM NaCl, 5% (v/v) glycerol, 1 mM ethylenediaminetetraacetic acid (EDTA), 10 mM L-arginine with 0.5% (v/v) membrane protease inhibitors (Sigma-Aldrich) and 1.5% (w/v) OGNG 40:1 CHS. Rho-1D4 antibody-coupled Sepharose beads were added after centrifugation, as above and washed with 50 column volumes (CV) 20 mM HEPES pH 7.5, 300 mM NaCl (wash buffer, WB) with 0.580% (w/v) OGNG 40:1 CHS, then 50 CV WB with 0.174% (w/v) OGNG 40:1 CHS. HHAT was eluted by overnight incubation with 3C protease. Elution was concentrated in Vivaspin 100,000 kDa molecular weight cut-off (MWCO) centrifugal concentrators (Sartorius) to a volume of ∼800 μL. The sample was passed over a Superose 6 Increase 10/300 GL column (GE Healthcare), equilibrated in 10 mM HEPES pH 7.5, 150 mM NaCl, 0.174% (w/v) OGNG 40:1 CHS. Protein-containing fractions were pooled and concentrated to 1 mg/mL.

#### Generation of nanobodies and megabodies

Nanobodies were constructed as described in Pardon et al. (2014). cDNA derived from Llama (*Lama glama*) serum was cloned into the pMESy4 (GenBank KF415192) phage display and bacterial expression vector. Megabody-expressing vectors were constructed as described in Uchański et al. (2021) via insertion of nanobody-encoding DNA into pMESP23E2 (GenBank: MT338521.1), which contains a circular permutant of *Escherichia coli* K12 glucosidase YgjK (UniprotKB: P42592; Gene ID: 947596) inserted into the first β-turn of NB169 or NB177.

Nanobodies and megabodies with C-terminal His_6_ tags were expressed in WK6 *E. coli* cells grown to an optical density of 0.7-1.2 in terrific broth supplemented with ampicillin at 37°C, 180 rpm. Expression was induced with 1 mM isopropyl β-d-1-thiogalactopyranoside (IPTG) and incubation temperature was lowered to 20°C overnight. Periplasmic extraction was performed in PE buffer (50 mM Tris pH 8.0, 150 mM NaCl, 20% (w/v) sucrose, 0.4 mg/mL lysozyme, DNase, 1 mM PMSF, protease inhibitor cocktail) and incubated at 4°C for 30 min with shaking. The soluble fraction was removed by centrifugation. Proteins were purified by nickel-affinity chromatography and samples were passed over a HiLoad 16/600 Superdex 75 or 200 pg column (GE Healthcare), equilibrated in 20 mM Tris pH 8.0, 300 mM NaCl. Protein-containing fractions were concentrated to 50-400 μM in Amicon Ultra concentrators (Merck-Millipore), before addition to HHAT to form complexes, except for the HHAT-IMP-1575-MB177 complex, where purified MB177 was flash-frozen and stored at −80°C.

#### Sonic Hedgehog (SHH) expression and purification

Wild-type sonic hedgehog (SHH) from *H. sapiens* (UniprotKB: Q15465, residues 24-193) was cloned into the pET22B bacterial expression vector, with a C-terminal His_6_ tag. SHH was expressed from *E. coli* BL21-DE3 cells using an adapted protocol described previously (Rudolf et al., 2019; Whalen et al., 2013). Cultures were grown in LB at 37°C, 180 rpm to an optical density of 0.6-0.8 before induction with 1 mM IPTG and decrease of temperature to 20°C overnight. Harvested cell pellets were resuspended in (1 x PBS, + 21 g/L NaCl, + 70 μL beta-mercaptoethanol, 1 mM PMSF, 1 protease inhibitor tablet). Cells were lysed using a cell disruptor and cell debris was removed via centrifugation. The protein was purified from the supernatant using TALON beads. The sample was then further purified using size exclusion chromatography (SEC) (HiLoad 16/600 Superdex 75 pg column, GE Healthcare) equilibrated in 5 mM Na_2_HPO_4_ pH 5.5, 150 mM NaCl, 0.5 mM dithiothreitol (DTT). Protein-containing fractions were concentrated and snap-frozen in liquid nitrogen for storage at −80°C.

#### Cryo-EM sample preparation, image collection and processing

Purified HHAT protein was mixed with the corresponding purified megabody at 1:1.5 molar ratio and purified by SEC using a Superose 6 Increase 10/300 GL column in 20 mM Tris, pH 8.0, 150 mM NaCl, at 4°C. The purified complex was concentrated to ∼4 mg/mL. For ligand-bound complexes, a final concentration of 5 mM nhPalm-CoA or 1 mM IMP-1575 inhibitor was added to the solution before grid plunging. A sample volume of 3.5 μL was placed on glow-discharged 300 copper mesh Quantifoil Holey Carbon grids R 1.2/1.3, before blotting for 3.0 s and flash-freezing in liquid ethane. All grids were prepared using a Vitrobot mark IV (FEI) at 4°C¸ 95%–100% humidity.

Cryo-EM data for the HHAT-MB177-nhPalm-CoA, HHAT-MB169-nhPalm-CoA and HHAT-MB177-IMP-1575 sample were collected on a 300 kV Titan Krios microscope (Thermo Fisher Scientific) at the Electron Bio-Imaging Centre (eBIC). Automated data collection was setup in EPU using a K3 (Gatan) direct electron detector operating in super-resolution mode with a physical pixel size of 0.829 Å/px (0.4145 Å/px super-resolution pixel) or 0.831 Å/px (0.4155 Å/px super-resolution pixel) and a GIF Quantum energy filter (Gatan) with 20 eV slit. Sample was collected with a total dose of ∼53-61 e-/Å2 across 50 frames. Sample-specific data collection parameters are summarized in Table S1.

Data processing pipelines are shown in Figure S2. Briefly, data were processed using cryoSPARC v.3.1.1 (Punjani et al., 2017) standard workflow. Raw movies were aligned with patch motion correction and binned 2 times at the motion correction step, giving a final pixel size of 0.829 Å/px or 0.831 Å/px. The contrast transfer function (CTF) was initially estimated using Patch-CTF. Poor-quality images were discarded after manual inspection. Particles were blob picked and the 2D classes were inspected and classes of interest were selected to generate templates for complete particle picking.

For the HHAT-MB177-nhPalm-CoA complex, a total number of 606,945 particles were picked and extracted in a 400 px box Fourier cropped to 200 px (1.662 Å/px). After template picking, 2D classification was skipped and 5 *ab initio* models were directly generated and further refined using heterogenous refinement, leading to a single good-looking class. Particles within this class with a 3D *ab initio* class posterior probability lower than 90% were discarded (39,331 particles) and the other particles were recentered and re-extracted in a 400 px box Fourier cropped to 300 px (1.1053 Å/px) before running a non-uniform refinement. The consensus map was refined to 3.0 Å. CTF refinement and high order aberration correction improved map resolution to 2.89 Å. Particles were exported to Relion 3.1.1 (Zivanov et al., 2018) using csparc2star.py and Bayesian polished (Asarnow et al., 2019). Focus refinement in cryoSPARC after particle subtraction was performed on the nanobody-HHAT part improving the resolution to 2.7 Å. A round of 3D classification without alignment in Relion with 10 classes was performed, leading to a major class with 238k particles. This class was further refined using focus refinement in cryoSPARC, and despite the same reported resolution, the quality of the map was significantly improved. In parallel, the particles were reverted to the original and a focus refinement after particles subtraction was performed on the megabody core, leading to a resolution of 2.6 Å. Both focus refined maps were combined using Phenix combine focused map followed by phenix autosharpen map tool (Adams et al., 2010). The combined map was used for model building after assessing that the quality of the combined map is identical to the focused maps.

For the HHAT-MB169-nhPalm-CoA complex, a total number of 2,300,000 particles were picked and extracted in a 400 px box Fourier cropped to 200 px (1.662 Å/px). Around 1,000,000 particles were selected after 2D classification and 5 *ab initio* models were then generated and further refined using heterogenous refinement, leading to a single good-looking class. Particles within this class with a 3D *ab initio* class posterior probability lower than 90% were discarded and the other particles were recentered and re-extracted in a 400 px box Fourier cropped to 300 px (1.1053 Å/px) before running a non-uniform refinement. The consensus map was refined to 3.4 Å. Particles were exported to Relion 3.1.1 (Zivanov et al., 2018) using csparc2star.py (Asarnow et al., 2019) and Bayesian polished before an extra step of 3D classification. The best-looking class containing 192K particles was further refined to 3.1 Å. Focus refinement on the nanobody-HHAT part greatly improved the quality of the transmembrane regions of HHAT with a final resolution of 3.7 Å.

Finally, for the HHAT-MB177-IMP-1575 complex, a total number of around 3,000,000 particles were picked and extracted in a 400 px box Fourier cropped to 200 px (1.62 Å/px). Around 864k particles were selected after 2D classification and 5 *ab initio* models were then generated and further refined using heterogeneous refinement, leading to two good-looking classes. Particles within these classes with a 3D *ab initio* class posterior probability lower than 90% were discarded and the other particles were recentered and re-extracted in a 400 px box Fourier cropped to 300 px (1.1053 Å/px) before running a non-uniform refinement. The consensus map containing around 367k particles was refined to 4.0 Å. After an extra step of 3D classification, the best-looking class containing 318K particles was further refined to 3.86 Å. Focus refinement on the nanobody-HHAT part greatly improved the quality of the transmembrane regions of HHAT with a final resolution of 3.59 Å.

#### Model building, refinement and validation

The structures were modeled by first fitting a homology model of HHAT and PDB entry 6XUX for the megabody component into the combined map of HHAT-MB177-nhPalm-CoA using UCSF Chimera (Pettersen et al., 2004). One cycle of rigid body real space refinement followed by manual adjustment in Coot (Emsley et al., 2010) was performed to correctly position the Cα chain into the density. Finally, cycles of Phenix (Adams et al., 2010) real space refinement and manual building in Coot (Emsley et al., 2010) were used to improve model geometry. Map-to-model comparison in Phenix mtriage validated that no over-fitting was present in the structures. Model geometry was validated for all models using MolProbity (Chen et al., 2010). This model was then used to refine the HHAT-MB169-nhPalm-CoA and HHAT-MB177-IMP-1575 complexes in a similar way. All map and model statistics are detailed in Table S1.

#### Coarse-grained molecular dynamics simulations

Ligands and the bound megabody were removed from the HHAT-MB177-nhPalm-CoA-heme structure and loops between D45-L51, W189-T197 and P409-Q422 modeled using Modeler 9.20 (https://salilab.org/modeler). The model with the lowest DOPE score was selected and converted to CG resolution using *matinize.py*. The MARTINI 2.2 forcefield (de Jong et al., 2013) was used to describe all components and the ElNeDyn elastic network applied to HHAT (force constant: 1000 kJ mol^-1^ nm^-2^, cut-off: 0.9 nm) (Periole et al., 2009). HHAT was positioned in a bilayer composed of POPC (35%), DOPC (35%), POPE (8%), DOPE (7%), cholesterol (10%) and palmitate (5%) in the luminal leaflet and POPC (15%), DOPC (15%), POPE (19%), DOPE (18%), POPS (8%), PIP_2_ (10%), cholesterol (10%) and palmitate (5%) in the cytoplasmic leaflet using *insane.*py (Wassenaar et al., 2015). This bilayer composition was selected to recapitulate the main features of ER membranes e.g., low sphingolipid content and a cholesterol:phospholipid ratio of approximately 0.15 (Jacquemyn et al., 2017). Palmitate was modeled with a charged headgroup (named PCN in the MARTINI topology) and cholesterol was modeled with inclusion of virtual sites. The membrane position around DltB (PDB ID.: 6BUG) on the MemProtMD server (memprotmd.bioch.ox.ac.uk) was used to guide HHAT insertion into the bilayer. The system was solvated using *insane.py* (Wassenaar et al., 2015) and approximately 0.15 M NaCl added to yield a total box size of 14 × 14 × 9 nm^3^. Prior to the production run, each replicate was independently energy minimized using a steepest decent method and equilibrated in two steps (step 1: 25 ns with all protein beads restrained, step 2: 100 ns with backbone beads restrained).

CG MD simulations (10x 15 μs) were performed using GROMACS 2019.4 (www.gromacs.org). The timestep was 20 fs and periodic boundary conditions were applied. Coulombic interactions were described using the reaction-field method and a 1.1 nm cut-off. Van der Waals interactions were described with the potential-shift Verlet method and a 1.1 nm cut-off. The V-rescale thermostat (Bussi et al., 2007) was used to maintain temperature as 310 K with a 1.0 ps coupling time constant. Pressure was maintained at 1 bar using the Parrinello-Rahman barostat, a 12.0 ps coupling time and a compressibility of 3x 10^−4^ bar^-1^.

#### Atomistic molecular dynamics simulations

A snapshot from the CG simulations was selected for backmapping to atomistic resolution using a revised CG2AT protocol (Vickery and Stansfeld, 2021) (https://github.com/owenvickery/cg2at). Prior to backmapping, palmitate was removed from the bilayer and a short equilibration performed to close gaps in the bilayer since topology files for backmapping palmitate were not available. The protein conformation was backmapped to the structural coordinates such that only the lipid arrangement reflected those of the CG snapshot. TIP3P water was readded to the system along with approximately 0.15 M NaCl.

The protein coordinates from the HHAT-nhPalm-CoA-heme structure were used to build the following systems: HHAT apo, HHAT with Palm-CoA bound and HHAT with Palm-CoA bound and heme covalently bound to Cys324. The protein coordinates from the HHAT-IMP-1575 structure were used to build the following system: HHAT in an inhibited conformation (but without inhibitor bound) with heme covalently bound to Cys324. The protonation state of residue sidechains in the input structures were assessed using propKa (https://github.com/jensengroup/propka-3.0) which did not identify any unusual pKas, therefore D339 was modeled as deprotonated and H379 was neutral. The CHARMM-36 forcefield (Huang and MacKerell, 2013) was used to describe all components. Palm-CoA parameters were derived from Stix et al. (2020). Standard CHARMM-36 parameters were used for the Mg ion bound to Palm-CoA (which remained bound without the need for additional restraints) and for the heme/Fe complex. To describe heme-Cys324 bonding, Cys324 was modeled as a thiolate (named CYM in the CHARMM-36 forcefield) and S-Fe bond parameters described identically to existing parameters for heme coordinated by methionine. Control simulations of heme without bonding to Cys324 were also performed, yielding similar results (data not shown). Each replicate was energy minimized using a steepest decent method then subject to 2x 5 ns NVT and NPT equilibration steps with position restraint applied to the protein backbone and the heavy atoms of bound ligands.

Each system was simulated for 5x 200 ns with a 2 fs timestep using GROMACS 2019.4 (www.gromacs.org). The Particle-Mesh Ewald (PME) method was used for long-range electrostatic interactions with a 1.2 nm cut-off. Van der Waals interactions were smoothly switched between 1.0 and 1.2 nm using the force-shift modifier. Temperature was maintained at 310 K using the Nosé-Hoover thermostat with a 0.5 ps coupling time. Pressure was maintained at 1 bar using the Parrinello-Rahman barostat, a 2.0 ps coupling time and a compressibility of 4.5 × 10^−5^ bar^-1^. A dispersion correction was not applied. The LINCS algorithm was used to constrain bonds to their equilibrium values.

In simulations initiated from the HHAT apo conformation a DOPC lipid bound within the luminal gate was removed after 200 ns in each of the 5 replicates. After lipid removal, each replicate was simulated for an additional 200 ns.

#### Steered molecular dynamics simulations

In addition to removal of the DOPC lipid (at t = 200 ns) from the luminal gate (described above) atomistic simulations were also performed where the lipid was pulled laterally into the membrane over a period of 10 ns before 40 ns of additional unbiased simulation time for each of the five replicates. Steered MD simulations were assisted by use of PLUMED (PLUMED Consortium, 2019). The collective variable used during steered MD was defined as the distance between the center of mass of the DOPC lipid and the Cα atom of Arg176. A moving restraint was applied over 10 ns to gradually increase the force constant to k = 1000 kJ mol^-1^ nm^-2^. This restraint was switched back to k = 0 kJ mol^-1^ nm^-2^ over the following 0.5 ns before a further 39.5 ns of unbiased simulation. All other simulations conditions are identical to those described above.

#### Simulation analysis

MDAnalysis (Michaud-Agrawal et al., 2011) (https://www.mdanalysis.org) was used for solvent and lipid density calculations. PropKaTraj (https://github.com/Becksteinlab/propkatraj) was used to calculate pKa values over the trajectory. PyMol (https://pymol.org/2/) and VMD (Humphrey et al., 1996) were used for visualization. Tunnels were calculated using the Caver3.0 plugin (https://caver.cz) implemented in PyMol.

#### Detection of HHAT binding to nanobodies NB169 and NB177

Cos-7 cells were infected with pHR-HHAT-mVenus-1D4, expressing full-length human HHAT C-terminally fused to mono-Venus. Once the stable cell line was established, cells were plated onto glass coverslips and incubated for 24 hours at 37°C and 5% CO_2_. All subsequent procedures were carried out at room temperature. Initially, cells were washed with PBS and fixed in 4% paraformaldehyde for 10 min. Fixed cells were then permeabilised with 0.1% Triton X-100/PBS for 10 min and blocked with 10% FBS/PBS for 1h. Where indicated, cells were incubated with 2.5 μM nanobody in PBS for 1h. This was followed by several PBS wash steps and incubation with anti-His antibody (#25B6E11, Genscript) at a dilution of 1:500 (v/v) for 1h in 0.1% FBS/PBS. Unbound primary antibody was removed with PBS washes and bound His antibody was recognized with an anti-rabbit AF555 antibody incubated for 1h in 0.1% (v/v) FBS/PBS. Final wash steps were followed by mounting the coverslips on microscopy slides using Vectashield with Dapi. Slides were viewed on a Leica DMi8 microscope with a 100x oil immersion objective (HC PL APO, NA 1.47) and a Leica EL6000 fluorescence illuminator. Images were taken with a Hamamatsu Orca Flash 4.0 V2 Camera and processed in Fiji ImageJ (Schindelin et al., 2012).

#### Size exclusion chromatography coupled with multi angle light scattering (SEC-MALS)

OGNG-solubilised HHAT at 1 mg/ml was loaded onto a Superose 6 10/300 GL column (GE Healthcare), equilibrated in 10 mM HEPES pH 7.5, 150 mM NaCl, 0.174% (w/v) OGNG 40:1 CHS, on a Shimadzu system with an inline Dawn HELEOS-II 8-angle light scattering detector (Wyatt). Scattering data were analyzed and molecular weight was calculated using ASTRA 6 software (Wyatt). Values of dn/dc = 0.1926 mL g^-1^ and ε_280_ 3.198 mL (mg cm)^-1^ were used to calculate HHAT molecular mass. Protein conjugate analysis was performed with dn/dc = 0.1270 mL g^-1^ for OGNG (Nie et al., 2021).

#### Bio-layer interferometry

All measurements were performed at 22°C using streptavidin (SA) biosensors in an Octet Red96e (both ForteBio). Biotinylated HHAT-Avi-1D4 was immobilised at 0.015-0.03 mg/mL in 10 mM HEPES pH 7.5, 150 mM NaCl, 0.03% DDM 40:1 CHS. For nanobody and megabody kinetic measurements, NB169, NB177, MB169 and MB177 were diluted into the same buffer at a 100 nM and a 1:3 dilution series created. For single-cycle kinetics, five 180 s association steps were each followed by 20 s dissociation steps and a final 600 s dissociation. For SHH binding tests, SHH samples were diluted into 10 mM HEPES, pH 7.5, 150 mM NaCl, 0.06% (w/v) DDM 40:1 CHS, 1 mM DTT, 5 mM CaCl_2_ with or without 5-10 mM nhPalm-CoA. Multi-cycle kinetic experiments were performed with association and dissociation steps of 300 s and a 1:1.5 SHH dilution series starting at 5 μM. Competition experiments were performed in a similar manner in a buffer containing 200 nM NB169 or MB177, with 300 s association and 600 s dissociation steps. IMP-1575 was added to a concentration of 10 μM where indicated. HHAT mutant studies were performed in the same manner with 0.015 mg/mL biotinylated HHAT mutants loaded and binding of 500 nM NB169 or 4 μM SHH. Loading was normalized to *wild-type* HHAT. Data were processed and analyzed using ForteBio Data Analysis software (version 11.1). All kinetic data were double-referenced. Single-cycle kinetic data binding constants were calculated by the fitting of a 1:1 binding model for megabodies and nanobodies using MATLAB (R2021a). Multi-cycle kinetic data binding constants for SHH were calculated by the fitting of a one to two state binding model using TraceDrawer (Ridgeview Instruments).

#### UV/Vis spectroscopy

UV/Vis spectra were recorded in a range of 220-800 nm at room temperature in a 96-well UV-Star Microplate (Greiner) using a CLARIOstar plate reader (BMG Labtech) with appropriate buffer blanks.

#### HHAT enzymatic activity using Acyl-cLIP

Biotinylated HHAT mutants and non-biotinylated HHAT mutants were analyzed separately. For both groups, 5 μL of purified (mutant) protein sample was mixed with 1.67 μL of 4X Laemmli loading buffer (Bio-Rad), then 5 μL was loaded onto an SDS-PAGE gel (Either 12% isocratic (Bio-Rad) or 4%–15% gradient acrylamide (Bio-Rad)) and run at 200 V for 35 min. The gel was stained with InstantBlue Coomassie protein stain (Abcam) and destained overnight. The gel was then imaged on an ImageQuant LAS 4000 (G.E. Electronics) and the obtained bands quantified using ImageJ (v. 1.53e) (Schindelin et al., 2012). From these band intensities, dilution factors to normalize each mutant to the lowest concentration sample (G287V) were calculated. All sample concentrations were then equalised using elution buffer (20 mM HEPES pH 7.5, 300 mM NaCl, 5% (v/v) glycerol, 0.03% (w/v) DDM 40:1 CHS, 0.5 mM 1D4 peptide). The equal concentration protein samples were split in two; one part was loaded onto an SDS-PAGE gel using the procedure described above and one part was used in Acyl-cLIP analysis of enzymatic activity as described below.

For the Acyl-cLIP assay, in a 384-well assay plate (Corning) each well was filled with 4 μL reaction buffer (100 mM MES, pH 6.5, 20 mM NaCl, 1 mM DTT, 1 mM tris(2-carboxyethyl)phosphine (TCEP), 0.1% (w/v) bovine serum albumin) and either 4 μL Palm-CoA solution (15 μM in reaction buffer) or another 4 μL reaction buffer (negative control). Master mix buffer was prepared by adding 10% (v/v) DDM buffer (20 mM HEPES pH 7.3, 350 mM NaCl, 5% (v/v) glycerol, 1% (w/v) DDM) to a 2 μM FAM-SHH peptide (synthesized in-house as previously described (Lanyon-Hogg et al., 2019)) solution in reaction buffer. The final master mix was then prepared for each (mutant) HHAT sample by adding 3 μL normalized protein solution to 100 μL of master mix buffer. 12 μL of HHAT-containing master mix was then distributed to each well and the fluorescence polarization (Excitation 480 nm, emission 535 nm) measured for 1 hour at 2 minute intervals on an EnVision Xcite 2104 (PerkinElmer). Enzymatic activity of each mutant was measured with (+ Palm-CoA) and without (- Palm-CoA) Palm-CoA and in quadruplicate.

The slope of the resulting curves was calculated for each individual well using Prism 9.1.2 (GraphPad) and each well was background subtracted by subtracting the average of its corresponding (- Palm-CoA) value. The background subtracted data were then normalized to wild-type activity and plotted in a bar chart using Prism.

### Quantification and statistical analysis

Replicate and error bar information for the acyl-cLIP data as well as statistical details from simulations are as reported within the figure legends.

## Data Availability

All data are available from the corresponding authors and/or included in the manuscript. Cryo-EM density maps with the corresponding atomic coordinates have been deposited in the Electron Microscopy Data Bank with accession codes EMD-13764 (for the HHAT-MB177-nhPalm-CoA complex), EMD-13860 (for the HHAT-MB177-IMP1575 complex) and EMD-13861 (for the HHAT-MB169-nhPalm-CoA complex). Corresponding atomic coordinates for the for the HHAT-MB177-nhPalm-CoA, HHAT-MB177-IMP1575 and HHAT-MB169-nhPalm-CoA complexes have been deposited in the Protein Data Bank with accession codes 7Q1U, 7Q6Z and 7Q70. This paper does not report original code. Any additional information required to reanalyze the data reported in this paper is available from the lead contact upon request.

## Supplemental information

Document S1. Figures S1–S8 and Table S1

Document S2. Article plus supplemental information
