# Supplementary material for: Structure, mechanism, and inhibition of Hedgehog acyltransferase
Source: Mol Cell. 2021 Dec 16;81(24):5025–5038.e10. doi: 10.1016/j.molcel.2021.11.018 (PMC8693861; doi:10.1016/j.molcel.2021.11.018)
Supplement: Document S1. Figures S1–S8 and Table S1 [file mmc1.pdf]

Molecular Cell, Volume *81*

Supplemental information

Structure, mechanism, and inhibition

of Hedgehog acyltransferase

Claire E. Coupland, Sebastian A. Andrei, T. Bertie Ansell, Loic Carrique, Pramod Kumar, Lea Sefer, Rebekka A. Schwab, Eamon F.X. Byrne, Els Pardon, Jan Steyaert, Anthony I. Magee, Thomas Lanyon-Hogg, Mark S.P. Sansom, Edward W. Tate, and Christian Siebold

Molecular Cell, Volume *81*

Supplemental information

Structure, mechanism, and inhibition of Hedgehog acyltransferase

Claire E. Coupland, Sebastian A. Andrei, T. Bertie Ansell, Loic Carrique, Pramod Kumar, Lea Sefer, Rebekka A. Schwab, Eamon F.X. Byrne, Els Pardon, Jan Steyaert, Anthony I. Magee, Thomas Lanyon-Hogg, Mark S.P. Sansom, Edward W. Tate, and Christian Siebold

Supplementary Figures S1-S8 and Table S1 – Coupland *et al.* “Structure, Mechanism and Inhibition of Hedgehog Acyltransferase”

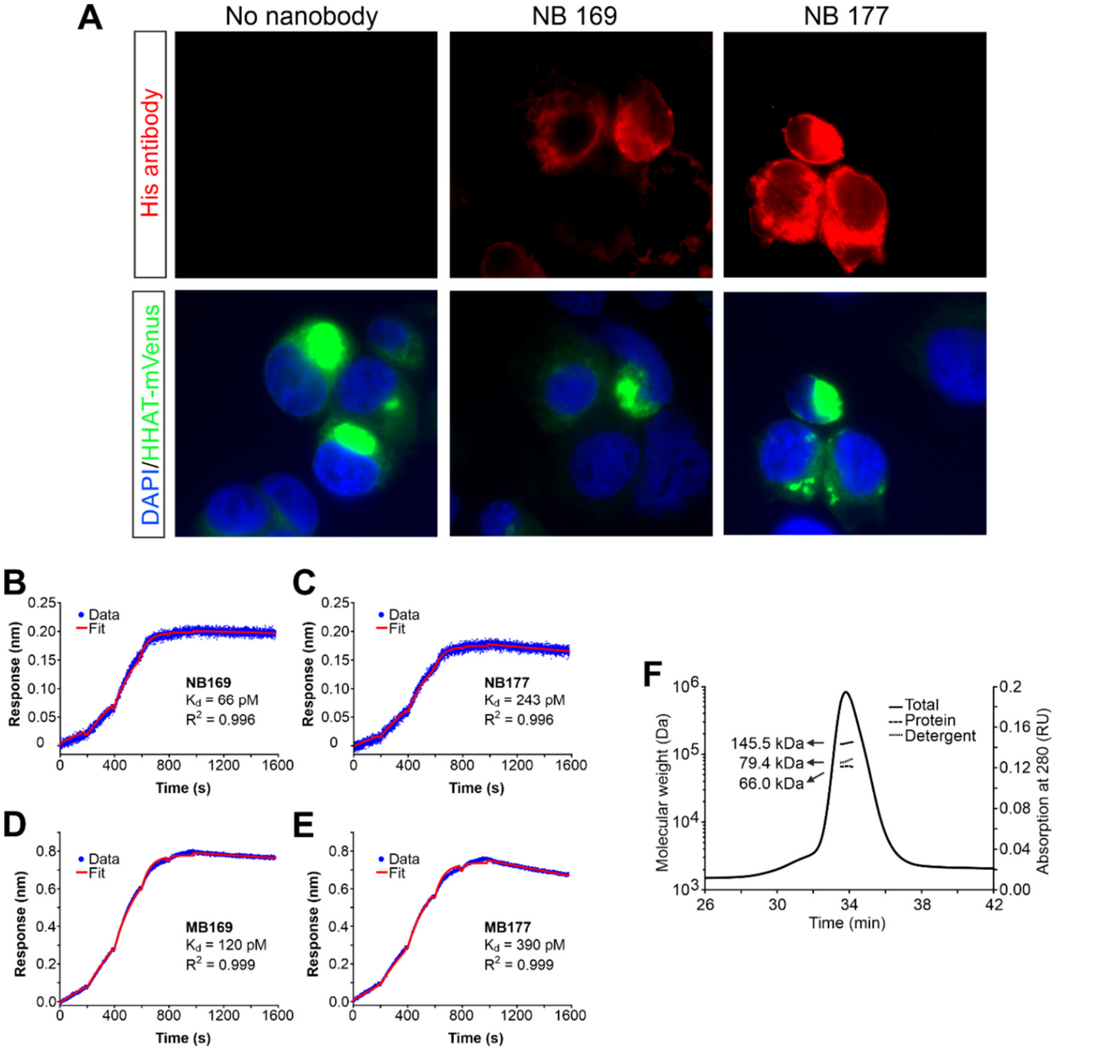

Fig. S1. Functional characterisation of HHAT-nanobody and -megabody interactions. Related to Fig. 1. (A) Immunofluorescence staining of mVenus-tagged full-length human HHAT stably expressed in COS-7 cells. The figure shows representative images of HHAT-mVenus expressing cells, incubated without or with either NB169 or NB177. Nanobodies were visualised by staining with primary and secondary antibodies and are shown in red, cell nuclei in blue and HHAT-mVenus in green. These analyses showed co-localisation between mVenus-tagged HHAT and NB staining, indicating that both nanobodies readily bind to membrane-embedded HHAT. **(B-E),** Biolayer interferometry (BLI) of HHAT-nanobody and -megabody interactions. Single cycle kinetic measurements for the HHAT-NB169 (**B**), HHAT-NB177 (**C**), HHAT-MB169 (**D**) and HHAT-MB177 (**E**) interactions are shown. K_d and R^2 are indicated for each measurement. **(F)** SEC-MALS analysis of OGNG-solubilised human HHAT. Molecular weights (MW, black lines) and 280 nm absorption (grey line) plotted against elution time. For clarity, graphs of MW are shown only around main absorption peak. Theoretical MW of HHAT based on sequence and including heme-B and 6 palmitoylation sites is 60.3 kDa. This analysis suggests that human HHAT is a monomer under our purification conditions.

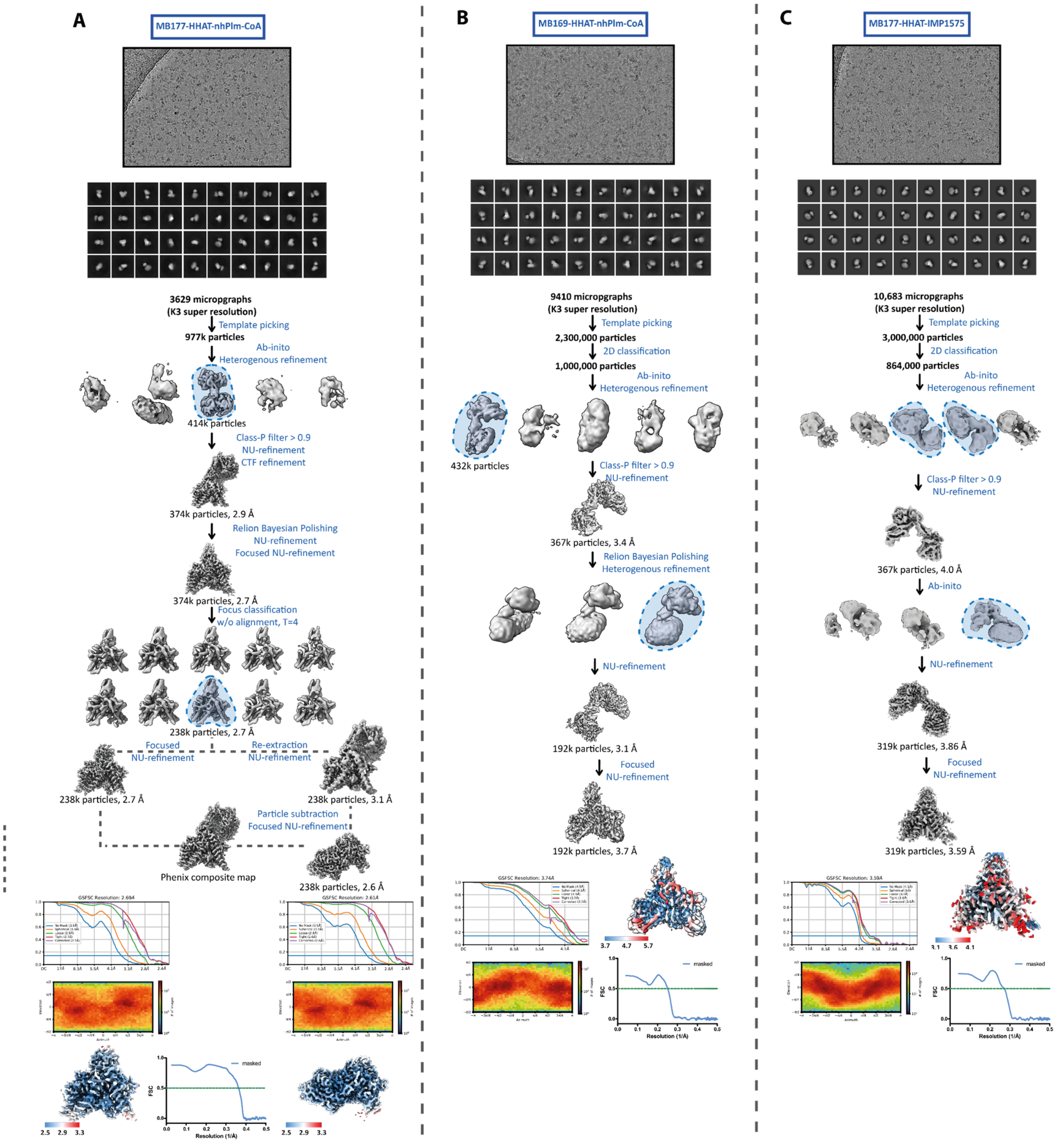

Fig. S2. Cryo-EM data collection, processing and analysis scheme. Related to Figs. 1-4. Flowchart for the processing and the classification of the HHAT-MB177-nhPalm-CoA (**A**, PDB ID 7Q1U), HHAT-MB169-nhPalm-CoA (**B**, PDB ID 7Q70) and HHAT-MB177-IMP-1575 (**C**, PDB ID 7Q6Z) complexes. For panels A-C, the top figure is a micrograph followed by 2D classes and the processing flowchart, the corresponding FSC plots indicating overall map resolution, the final angular distributions, the 3D reconstructions locally filtered and coloured according to local resolution and model-to-map FSC curves.

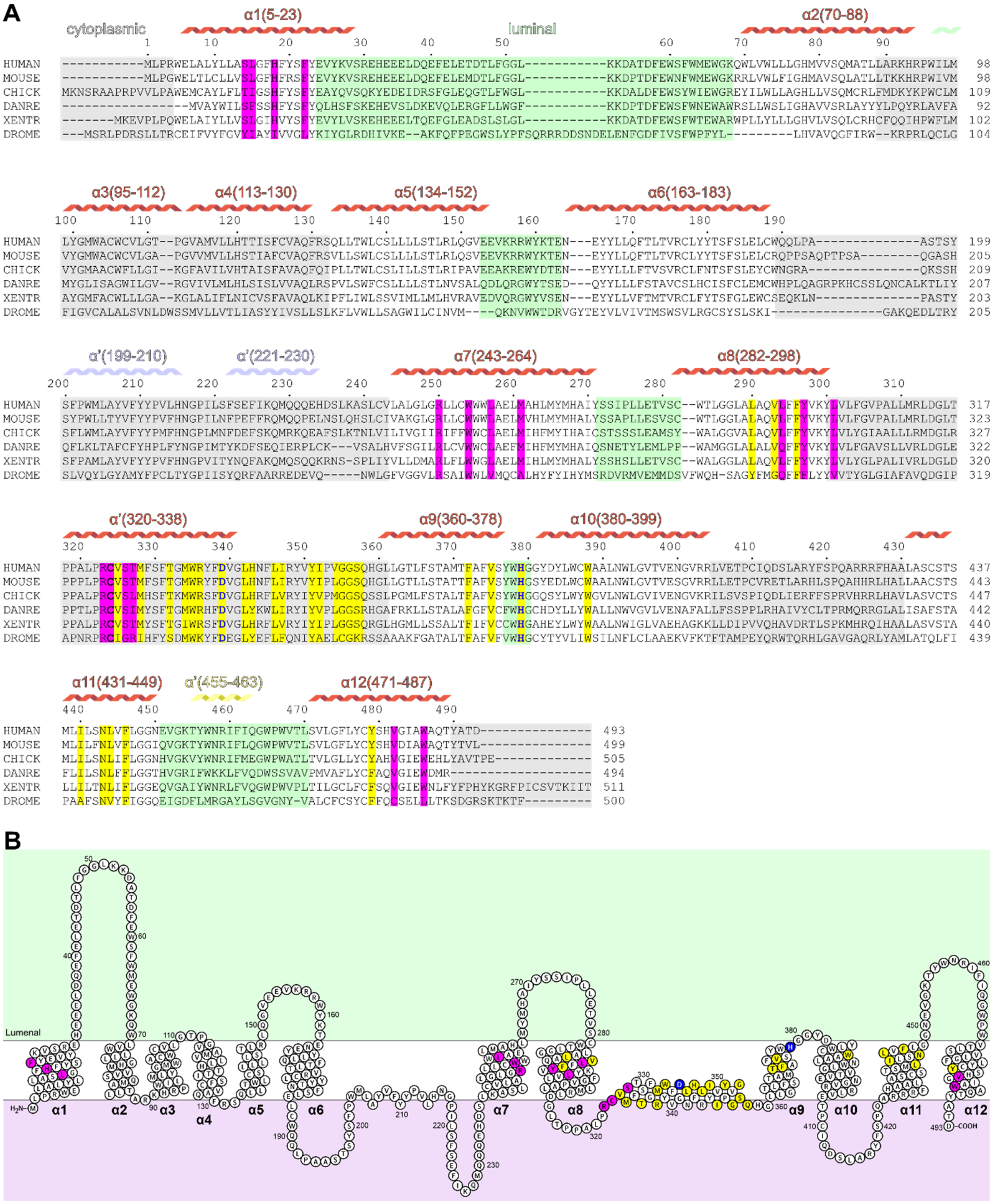

Fig. S3. Sequence alignment and topology of HHAT. Related to Figs. 1-3. (A) Sequence alignment of selected HHAT family members. Secondary structure assignment is displayed above the alignment. Grey boxes correspond to cytosolic and green boxes to ER luminal loop regions. Residues interacting with heme-B are in magenta and with nhPalm-CoA in yellow. Active site residues Asp339 and His379 are highlighted in blue. **(B)** Topology diagram of human HHAT adapted from the Protter server (wlab.ethz.ch/protter/start/) and colour-coded as in **A**. UniProt Ids are as follows: Q5VTY9 (human), Q8BMT9 (mouse), F1NHW1 (chick), F8W2J4 (zebrafish, DANRE), F6YU00 (frog, XENTR), Q9VZU2 (fly, DROME).

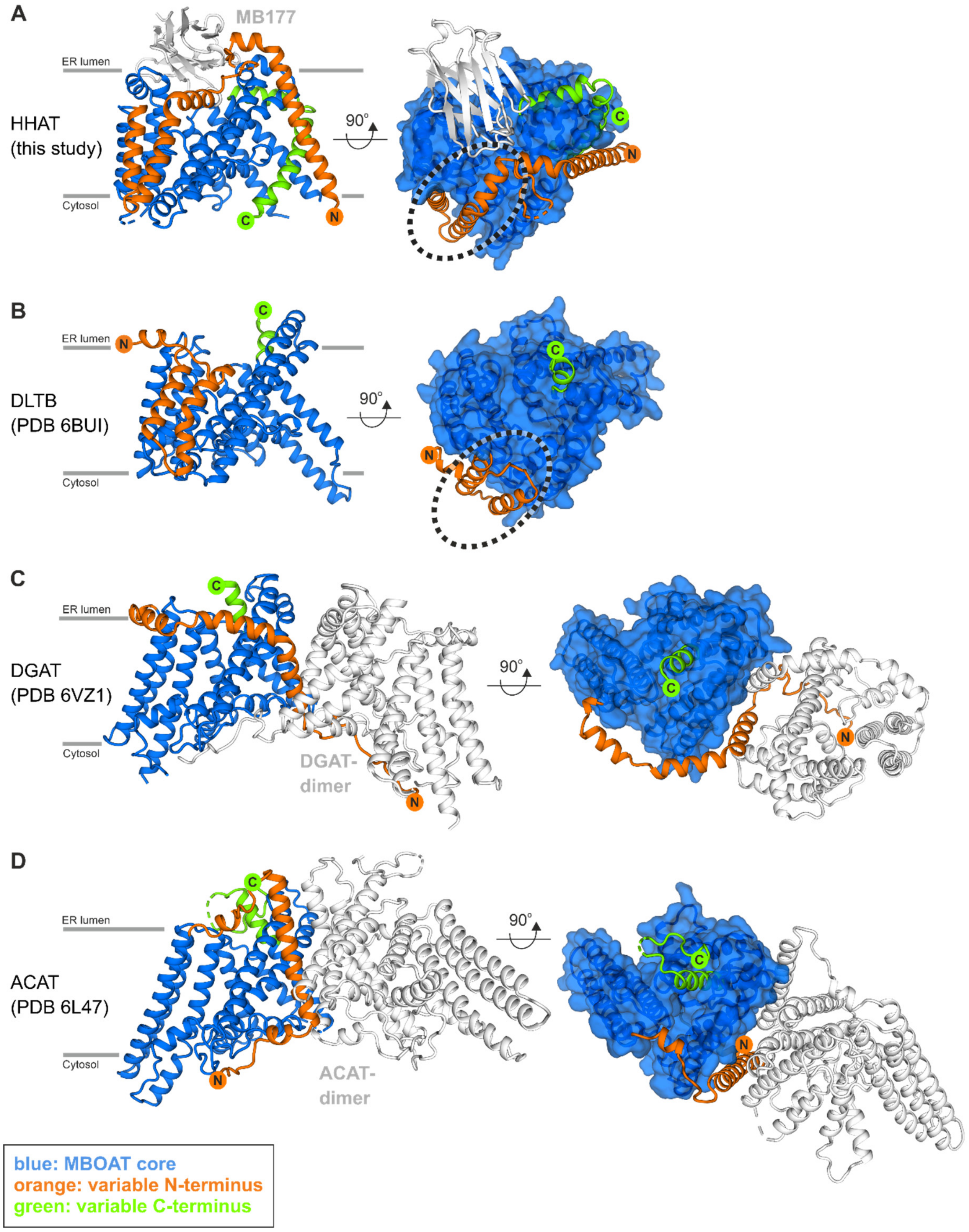

Fig. S4. Structural comparison of MBOAT superfamily members. Related to Fig. 1. Cartoon representations of HHAT (**A**), DLTB (**B**, PDB ID. 6BUI), DGAT (**C**, PDB ID. 6VZ1) and ACAT (**D**, PDB ID. 6L47). DLTB, DGAT and ACAT were superimposed onto HHAT. The conserved MBOAT core is coloured in blue, and the variable N- and C-termini in orange and green, respectively. The dotted circles in the right panels of **A** and **B** indicate conserved transmembrane helices in HHAT and DLTB, that block lipid-substrate access observed for DGAT and ACAT. These helices are rearranged in DGAT and ACAT to participate in dimer formation and allow access of substrates.

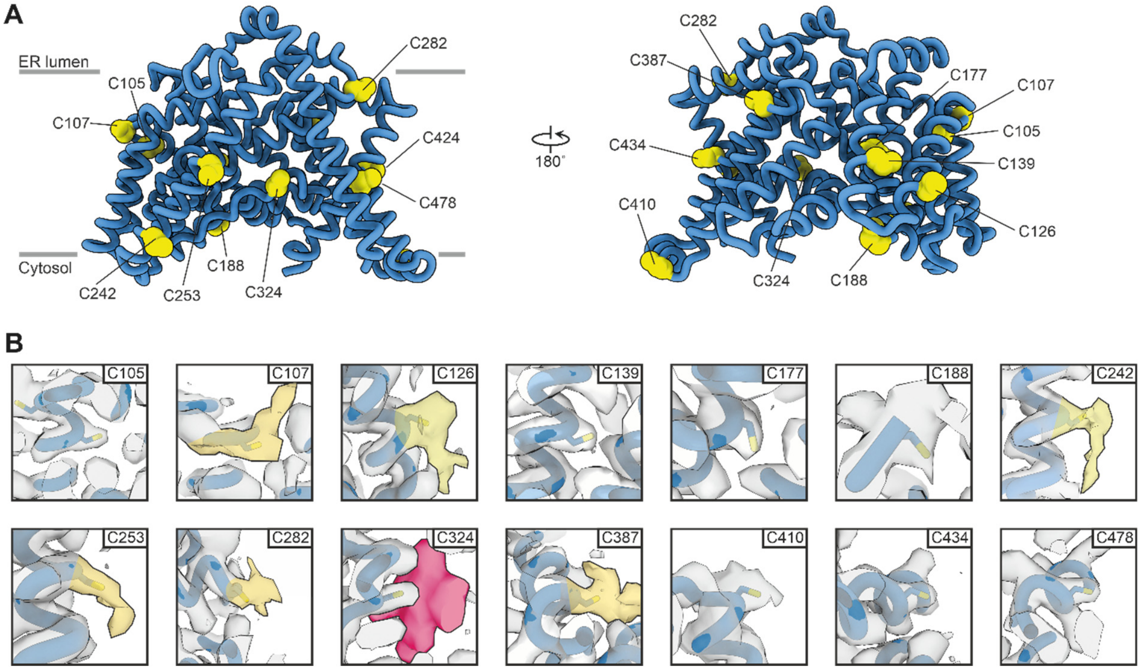

Fig. S5. Analysis of potential HHAT cysteine palmitoylation sites. Related to Fig. 1. (A) Cartoon representation of HHAT with all cysteine residues labelled and highlighted as yellow spheres. **(B)** Close-up views of all cysteine residues. The high resolution cryo-EM map is shown in grey. Observed additional density is highlighted in yellow. The density around side chain C324 that we identified as a heme-B group is depicted in red. We note that C188 is the terminal residue of a disordered, cytoplasmic loop and thus identification of additional map features that could correspond to palmitoylation are difficult to access.

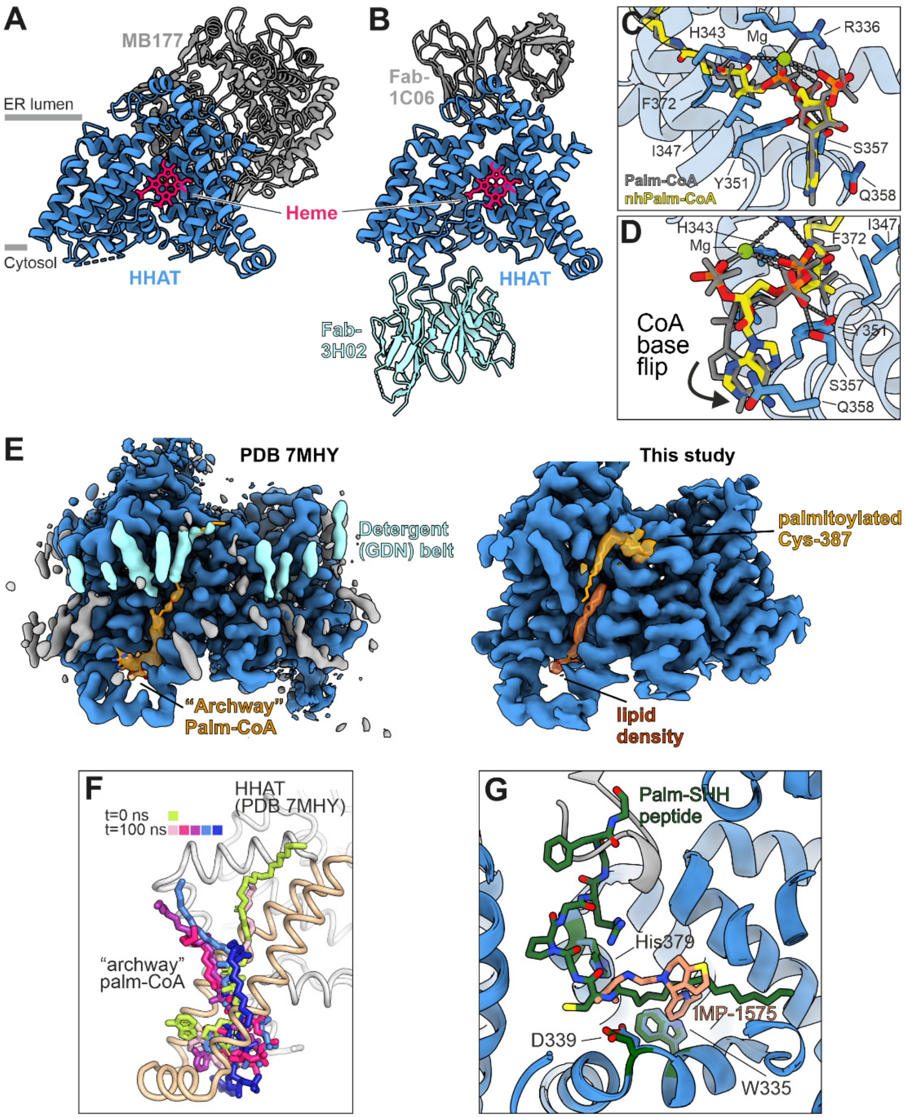

Fig. S6. Structural comparison to the HHAT-Fab1C06-Fab3H02 complex. Related to Figs. 1-3. (A, B) Cartoon representations of the HHAT-MB177 complex (this study) and the HHAT-Fab1C06-Fab3H02 complex (PDB ID. 7MHY). The cryo-EM maps obtained in both studies are of similar quality with an almost identical overall fold of HHAT (rmsd: 0.62 Å for 468 equivalent Cα positions) and a conserved binding mode for the previously uncharacterized heme group coordinated by Cys324. **(C, D)** Two close-up views of the Palm-CoA base region. nhPalm-CoA (this study) is shown in atomic colouring (yellow: carbon, red: oxygen, blue:nitrogen, orange: phosphor), the Palm-CoA (PDB ID. 7MHY) in grey. The movement of the base region is indicated with an arrow. (**E**) Map comparison of the "archway" Palm-CoA region between Jiang *et al.* (**left**, EMD-23836) and this study (**right**). The detergent (GDN) belt is coloured in cyan, the "archway Palm-CoA" in light orange and the palmitoylated C387 in dark orange. **(F)** Comparison of the position of the "archway" Palm-CoA as modelled by Jiang *et al.* (t = 0 ns, lime) or after 5 x 100 ns atomistic simulations (t = 100 ns, blues/purples). Simulations were performed using the HHAT structure from Jiang *et al.* bound to heme and both Palm-CoA molecules (PDB ID: 7MHY). Palm-CoA is shown as sticks bound within the proposed "archway" (light brown) shown in cartoon representation. **(G)** Close-up view of the reaction centre. Superposition of the HHAT-Palm-SHH peptide (green, Jiang *et al*, PDB ID. 7MHZ) and HHAT-IMP-1575 (blue/salmon, this study) complexes. Both IMP-1575 and the Palm-SHH peptide induce a similar rearrangement of HHAT active site residues H379 and D339, and "gate keeper" residue W335.

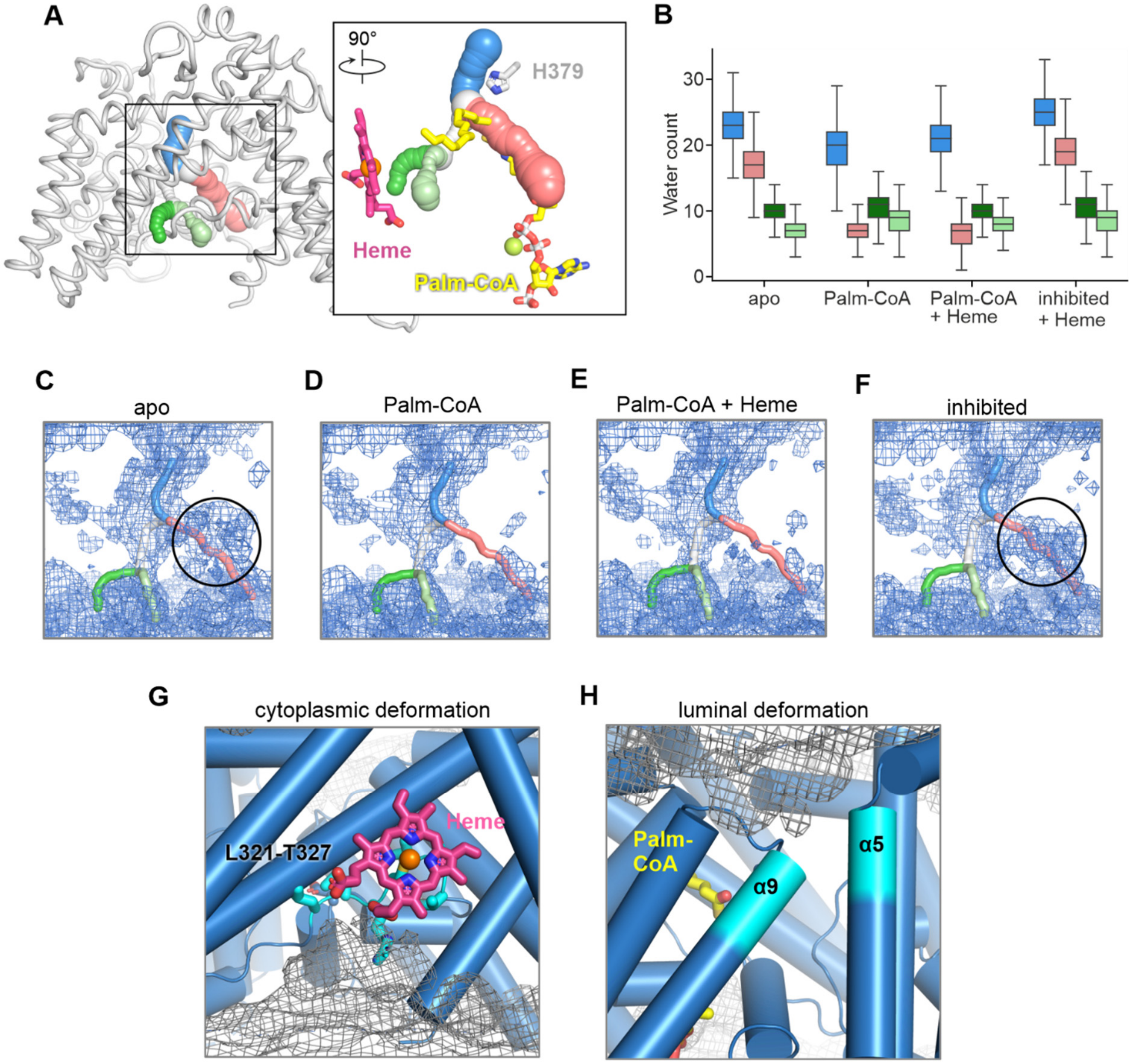

Fig. S7. Water and membrane densities in HHAT simulations. Related to Fig. 2. (A) Tunnels through the HHAT core as calculated using Caver. Tunnels are displayed as spheres. The inset indicates the position of tunnels with respect to the bound heme (pink), Palm-CoA (yellow) and His379 (grey) which are displayed as sticks. The Fe and Mg ions are shown as spheres in orange and green, respectively**. (B)** Quantification of the number of water within 4 Å of the centroid coordinates through the tunnels indicated in **A** across the final 50 ns of atomistic MD simulations of HHAT in the apo, Palm-CoA, Palm-CoA plus heme and inhibited/heme bound states. Boxplots are coloured according to the tunnel colours in **A**. **(C-F)** Time averaged solvent density within the HHAT core across atomistic simulations of HHAT in the apo (**C**), Palm-CoA bound (**D**), Palm-CoA plus heme (**E**) and inhibited/heme bound (**F**) conformations. Trajectories were combined and fitted before the density calculations which were performed using MDAnalysis. The centroid through the tunnels are shown as sticks and the circles indicates solvent within the Palm-CoA binding pocket. **(G-H)** Closeup of the regions of membrane deformation in the heme binding cavity and around α5/α9 (the luminal gate). Cyan sticks depict the location of a loop formed by Leu321-Thr327 which includes the Heme coordinating Cys324. Membrane density (grey isomesh) was calculated in CG simulations of HHAT and overlayed with the atomistic structure for reference.

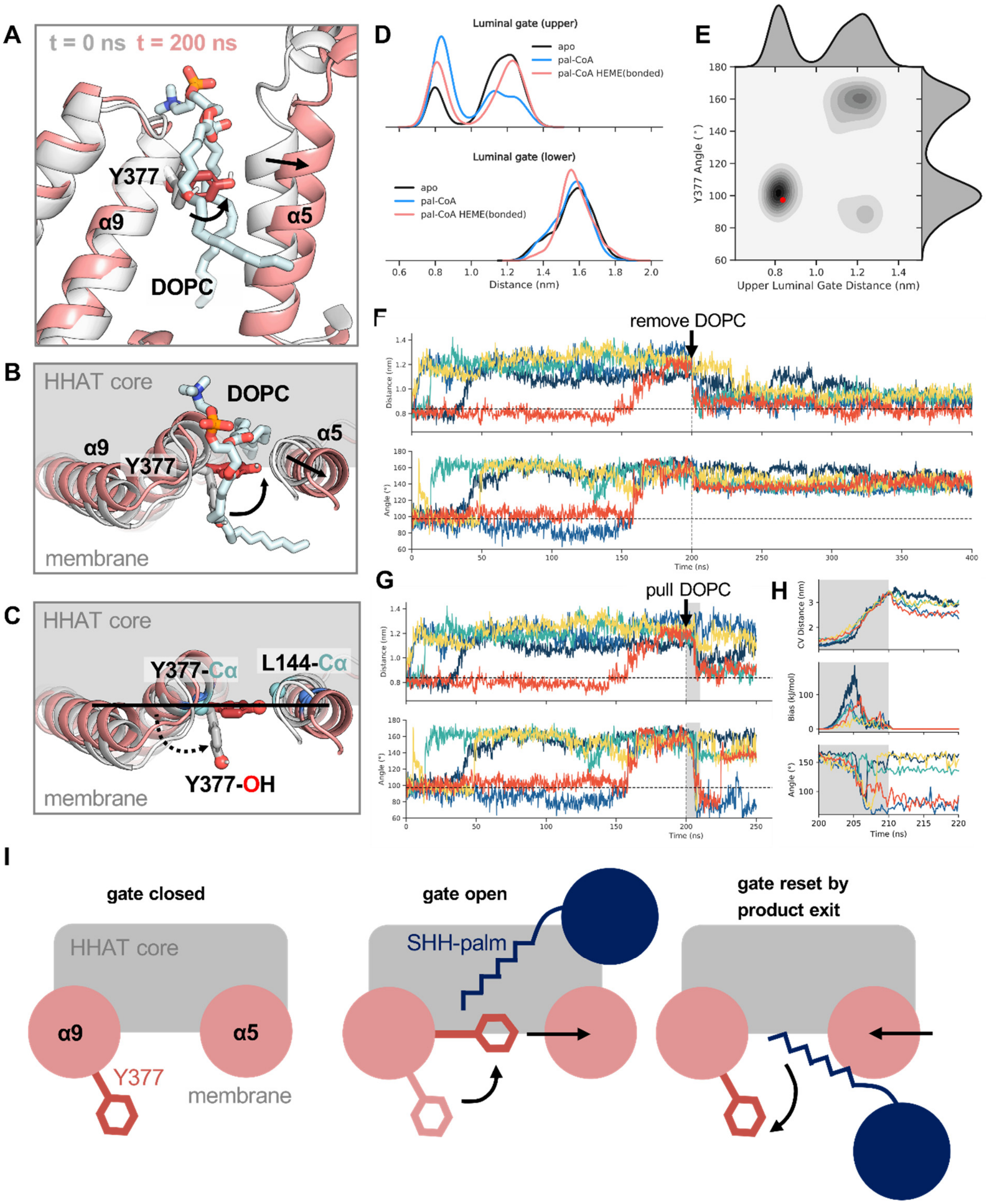

Fig. S8. A luminal exit gate for palmitoyl-SHH. Related to Fig. 1, 2 and 5. (A) Snapshots from the start (t = 0 ns, grey) and end (t = 200 ns, red) of an atomistic simulation of HHAT, with a DOPC lipid tail (light blue sticks) located in the luminal gate mimicking palmitoylated-SHH product. Arrows indicate outward movement of α5 and inward swivel of the Tyr377 sidechain (shown in stick representation). Atomistic simulations were initiated from a coarse-grain simulation snapshot where DOPC spontaneously and stably bound within the luminal gate (**Movie S1**). Backmapping to atomistic resolution was achieved using CG2AT (https://github.com/owenvickery/cg2at). **(B)** Top view of the luminal surface showing expansion of the luminal gate and change to the sidechain angle of Tyr377 when one acyl-tail of the DOPC lipid is positioned within the reaction centre. **(C)** Definition of the upper luminal gate distance and Tyr377 sidechain angle distributions analysed in **D-H**. The upper luminal gate distance was defined as the distance between Cα atoms of Try377/Leu144 (cyan spheres). The Tyr377 sidechain angle was

defined as the angle between a vector formed by the Cα and Oh (hydroxyl) atoms of Tyr377 and a vector formed by the Cα atoms of Tyr377/Leu144 (shown as spheres). **(D)** Distribution of distances at the top/bottom of α5/α9 across 5 x 200 ns atomistic simulations of apo HHAT (black), HHAT with palm-CoA bound (blue) and HHAT with heme and palm-CoA bound (red). All simulations were initiated with DOPC bound in the luminal gate indicating consistent opening of the top of the luminal exit gate due to outward movement of α5. **(E)** 2D correlation plot of the upper luminal gate distance vs the Tyr377 sidechain angle (as defined in **C**) across 5 x 200 ns simulations of apo HHAT. The red dot indicates the distance and angle at t = 0 ns. **(F)** Upper luminal gate distance and Tyr377 sidechain angle with time indicating correlated motions across the first 5 x 200 ns of apo HHAT simulations. At 200 ns the bound DOPC was removed from the luminal gate and a further 200 ns of atomistic simulation performed for each replicate to allow relaxation of the gate. **(G)** As in F but at 200 ns the DOPC was pulled out of the luminal gate into the membrane over a period of 10 ns (grey box) before a further 40 ns of unbiased atomistic simulation was performed for each replicate. The collective variable (CV) used during the steered MD simulations was defined as the distance between the centre of mass of the DOPC lipid and the Cα atom of Arg176. A force constant of k = 1000 kJ mol^{-1} nm^{-2} was gradually increased over the 10 ns steered MD simulation using PLUMED (https://www.plumed.org). **(H)** Zoomed in view of the steered MD simulation (grey box) from **G** indicating exit of DOPC through the gate as shown by the CV distance (top) as the work bias factor is increased (middle) and the Tyr377 sidechain angle swings outward (bottom) to permit DOPC exit. **(I)** Model of the proposed luminal exit gate mechanism for palmitoylated-SHH. In the absence of palmitoylated-SHH product the luminal gate formed by α5/α9 is closed. When palmitoylated-SHH is located within HHAT, the upper region of α5 tils outwards allowing the sidechain of Tyr377 to swing inwards and plug the gate. As palmitoylated-SHH moves laterally into the membrane the Tyr377 sidechain is pushed outwards, enabling inward movement of α5 to reset the luminal gate.

Table S1. Cryo-EM data collection and structure refinement. Related to Figures 1-3.

	**HHAT-MB177-nhPalm-CoA** EMD-13764 PDB ID 7Q1U	**HHAT-MB169-nhPalm-CoA** EMD-13861 PDB ID 7Q70	**HHAT-MB177-IMP1575** EMD-13860 PDB ID 7Q6Z
**Data Collection**			
**Microscope**	Titan Krios IV (eBIC)	Titan Krios IV (eBIC)	Titan Krios I (eBIC)
**Voltage (kV)**	300	300	300
**Detector**	Gatan K3 with EF	Gatan K3 with EF	Gatan K3 with EF
**Recording mode**	Super Resolution	Super Resolution	Super Resolution
**Magnification**	105,000	105,000	105,000
**Movie/micrograph pixel size (Å)**	0.4145	0.4145	0.4155
**Dose rate (e^-/px/sec)**	15.1	13.9	12.3
**Number of frames per movie**	50	50	50
**Movie Exposure time (s)**	2.5	3	3
**Total dose (e^-/Å^2)**	54.76	60.6	53.48
**Defocus range (μm)**	1.0 to 2.5	1.0 to 2.5	1.0 to 2.5
**Volta Phase Plate**	no	no	no
**EM Data Processing**			
**Number of movies/micrographs**	3637	9410	10,683
**Box size (px)**	400	400	400
**Particle number (total)**	977K	2,300,000	3,000,000
**Particle number (post 2D)**	/	1,003,000	864K
**Particle number (post 3D)**	414K	367K	319K
**Particle number (for final map)**	238K	192K	234K
**Symmetry**	C1	C1	C1
**Map resolution (FSC 0.143)**	2.7	3.74	3.65
**Local resolution range (FSC 0.5)**	2.45^{-6}	3.71^{-10}	3.07^{-6}
**Map sharpening B-factor (Å^2)**	-56	-125	-152
**Model Building and Validation**			
**Initial Model Used**	De novo	XXXX	XXXX
**Model Composition**			
Non-hydrogen protein atoms	21974	10178	9887
Protein residues	1357	606	597
Ligands			
nhPalmCoA	1	1	/
IMP-1575	/	/	1
Heme-b	1	1	1
Cholesterol	1	1	1
Palmitate	4	1	1
Mg^{2+}	1	0	0
**RMSD from ideal**			
Bond length (Å)	0.003	0.004	0.003
Bond angles (°)	0.558	0.663	0.593
**Validation**			
Molprobity score	1.39	1.92	1.51
Clashscore	4.42	11.47	6.39
Rotamers outliers (%)	0.61	0	0
FSC (0.5) model-vs-map	2.7	4	3.7
CC model-vs-map (masked)	0.8	0.65	0.71
**Ramachandran plot**			
Favoured (%)	97.03	95	97.11
Allowed (%)	2.97	5	2.89
Outliers (%)	0	0	0
